# Discovery of Crinasiadine, Trisphaeridine, Bicolorine, and Their Derivatives as Anti-Tobacco Mosaic Virus (TMV) Agents

**DOI:** 10.3390/ijms26031103

**Published:** 2025-01-27

**Authors:** Zhan Hu, Jincheng Guo, Dejun Ma, Ziwen Wang, Yuxiu Liu, Qingmin Wang

**Affiliations:** 1 Key Laboratory of Green Prevention and Control of Tropical Agriculture and Forestry BioDisasters of Ministry of Education, School of Tropical Agriculture and Forestry, Hainan University, Haikou 570228, China; huzhan@hainanu.edu.cn; 2 State Key Laboratory of Elemento-Organic Chemistry, Research Institute of Elemento-Organic Chemistry, Frontiers Science Center for New Organic Matter, College of Chemistry, Nankai University, Tianjin 300071, China; gjcheng@brire.com (J.G.); green9012@126.com (D.M.); hxxywzw@tjnu.edu.cn (Z.W.); liuyuxiu@nankai.edu.cn (Y.L.); 3 Baotou Research Institute of Rare Earths, Baotou 014030, China

**Keywords:** natural product, Amaryllidaceae alkaloids, crinasiadine, tobacco mosaic virus, antiviral activity, mechanism research

## Abstract

Plant viral diseases cause great harm to crops in terms of yield and quality. Natural products have been providing an excellent source of novel chemistry, inspiring the development of novel synthetic pesticides. The Amaryllidaceae alkaloids crinasiadine (**3a**), trisphaeridine (**4a**), and bicolorine (**5a**) were selected as parent structures, and a series of their derivatives were designed, synthesized, and investigated for their anti-plant virus effects for the first time. Compounds **13b** and **18** exhibited comparable inhibitory activities to ningnanmycin against tobacco mosaic virus (TMV). Preliminary research into the mechanism, involving transmission electron microscopy and molecular docking studies, suggests that compound **18** may interfere with the elongation phase of the TMV assembly process. This study provides some important information for the research and development of agrochemicals with phenanthridine structures.

## 1. Introduction

The growing global population and the decreasing amount of arable land make agricultural productivity a vital challenge to address [1,2,3]. Plant viruses, which are among the most devastating pathogens, cause enormous losses to agricultural industries worldwide and continue to threaten global food security [4]. Tobacco mosaic virus (TMV), one of the most important plant viruses, is known to infect at least 100 different plant species, including tobacco, tomatoes, peppers, cucumbers, and a number of ornamental flowers [5,6,7]. Unfortunately, few chemical agents have been commercialized as effective products against this plant virus [8,9]. Therefore, there is a critical need to discover and develop agents with different scaffolds or modes of action.

**Figure 1:**
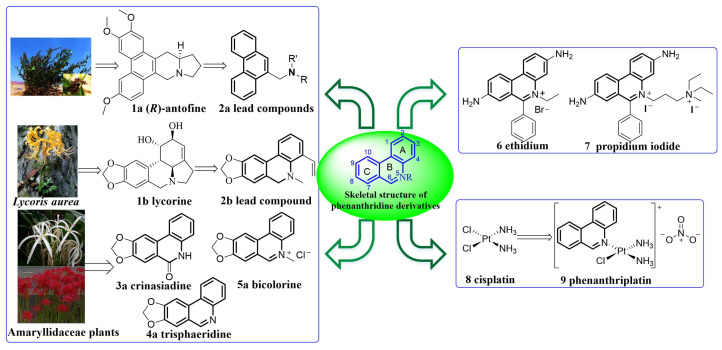
Structures of compounds **1a**, **1b**, **2a**, **2b**, **3a**~**5a**, and **6**~**9**.

It is well known that natural products provide an excellent source of novel chemistry and have inspired the development of novel synthetic pesticides [10,11]. In the process of discovering and modifying natural products as anti-plant virus agents, our research group first found that tylophorine alkaloids—especially antofine (**1a**, Figure 1)—had excellent anti-TMV activities, and the structure–activity relationship studies indicated that the phenanthrene ring and the nitrogen in the tertiary amine (**2a**, Figure 1) were essential for high antiviral activity [12,13]. Subsequently, we have optimized a multi-chiral natural product lycorine (**1b**, Figure 1) to a simple phenanthridine analog (**2b**, Figure 1) with good agricultural activities [14].

Indeed, phenanthridine analogs (crinasiadine (**3a**), trisphaeridine (**4a**), and bicolorine (**5a**), Figure 1), as natural products, are present in many Amaryllidaceae plants [15,16], and they have been one of the most widely studied classes of compounds due to their biological activities [17,18]. As early as the 1930s, phenanthridinium compounds were discovered to resist trypanosomes, and the anti-trypanosome drugs Ethidium and Samorin were developed [19,20,21]. To date, these two phenanthridinium trypanocides are still widely used to treat African trypanosomiasis in livestock [21,22,23]. Ethidium bromide (**6**, Figure 1) can also be used as a fluorescent marker for DNA and RNA, and its derivative propidium iodide (**7**, Figure 1) can be used as a cell viability probe [17]. Recent studies have found that phenanthridine analogs can inhibit the replication of the hepatitis C virus (HCV) [23,24] and the porcine epidemic diarrhea virus (PEDV) [25], as well as act as an activator of the Wnt/*β*-catenin signaling pathway [26,27,28,29,30]. When one chlorine atom of the commercially available anticancer drug cisplatin (**8**, Figure 1) was replaced with phenanthridine, the new compound phenanthriplatin (**9**, Figure 1) was more likely to enter cancer cells with higher efficacy and fewer side effects [31,32]. Due to their interesting biological activity, several efforts have been made to prepare phenanthridine derivatives [18,33].

In order to continue our previous research [14] and further explore the agricultural activities of phenanthridine compounds, in this work, crinasiadine (**3a**), trisphaeridine (**4a**), and bicolorine (**5a**) were selected as parent structures, and a series of their derivatives were designed, synthesized, and evaluated for their anti-TMV activities. In addition, the preliminary mode of action of compound **18** against the TMV was also explored.

## 2. Results and Discussion

### 2.1. Chemistry

**Scheme 1:**
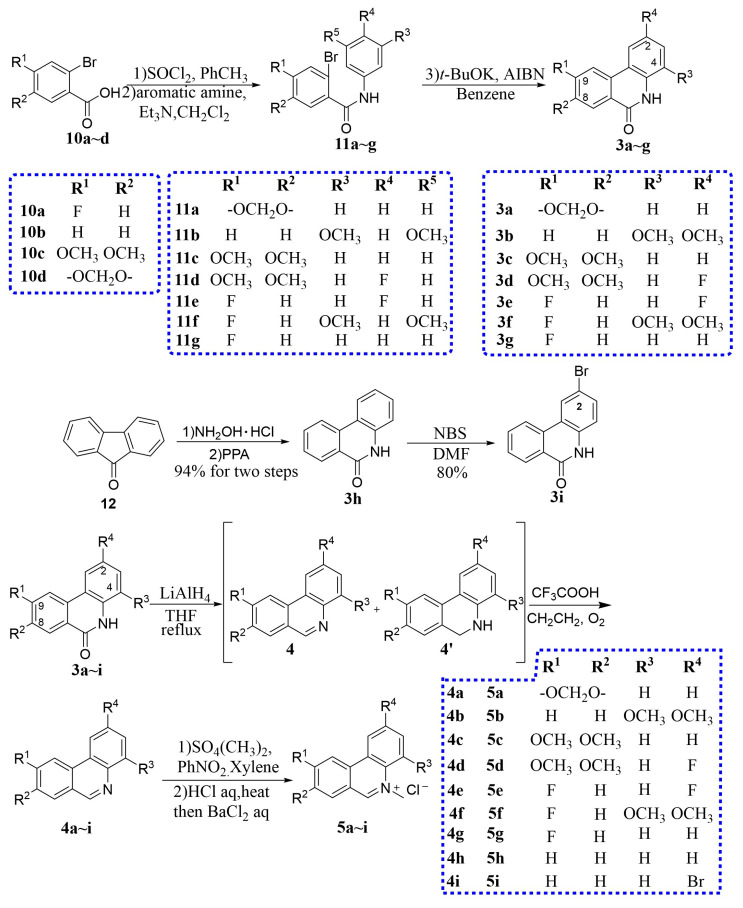
Synthetic route of the target compounds **3**, **4**, and **5**.

**Scheme 2:**
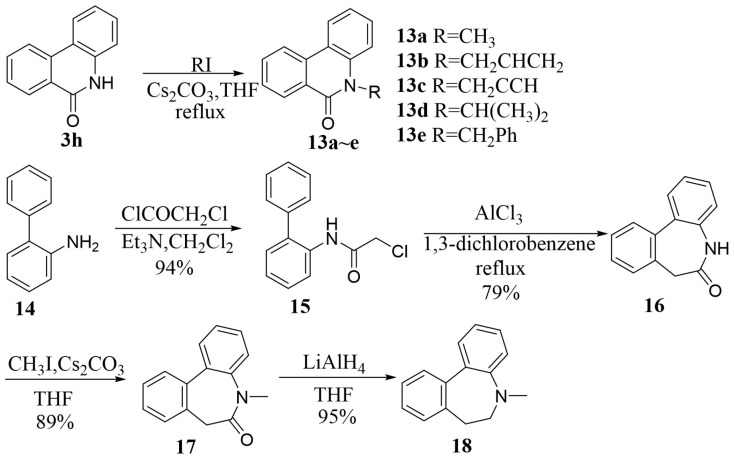
Synthetic route of the target compounds **13**~**18**.

In this study, 35 diverse structures of phenanthridine analogs including phenanthridin-6(5*H*)-one derivatives (**3a**~**i**, **13a**~**e**), phenanthridine derivatives (**4a**~**i**), 5-methylphenanthridinium chloride derivatives (**5a**~**i**) and phenanthridone B-ring-expanded derivatives (**16**~**18**) were synthesized (Scheme 1 and Scheme 2). The synthetic routes are given in Scheme 1 and Scheme 2. There are fewer substituents on the benzene ring of the natural products crinasiadine (**3a**), trisphaeridine (**4a**) and bicolorine (**5a**). We synthesized phenanthridine compounds with a common electron-withdrawing group (F, Br) or electron-donating group (OCH_3_) on the benzene ring as compensation. Phenyl ring-substituted phenanthridin-6(5*H*)-ones (**3a**~**3g**) were synthesized from 2-bromo-*N*-phenylbenzamides (**11a**~**g**) in the presence of *t*-BuOK and a catalytic amount of AIBN through intramolecular free radical reactions [34]. 2-Bromo-*N*-phenylbenzamides (**11a**~**g**) were obtained with high yields from 2-bromobenzoic (**10a**~**d**) and phenyl anilines [34]. Phenanthridin-6(5*H*)-one (**3h**) was easily synthesized from 9-fluorenone (**12**) via Beckmann rearrangement [35]; then, compound **3h** [36] reacted with NBS to give compound **3i** with good yield. Phenanthridin-6(5*H*)-one derivatives (**3**) can be reduced by LiAlH_4_ to mainly give compound **4′** and a small quantity of compound **4**. However, compound **4′** was unstable and easily converted into complex compounds when separated with a normal pressure silica gel column. We found that compound **4′** could be aromatized to phenanthridine derivatives (**4**) with good yield under oxidizing and acidic environmental conditions. Therefore, the reduction products of phenanthridone analogs (**3a**~**i**) without isolation were directly dissolved in CH_2_Cl_2_ with an amount of CF_3_COOH and then stirred with air bubbling to convert them to phenanthridine derivatives (**4a**~**i**). With the phenanthridine analogs (**4a**~**i**) in hand, it was easy to prepare 5-methylphenanthridinium chloride (**5**) via dimethyl sulfate according to the procedures reported previously [37]. A mixture of the phenanthridin-6(5*H*)-one (**3h**), Cs_2_CO_3_, haloalkanes, and anhydrous THF was gently refluxed to give compounds **13a**~**e** with good yield [38]. B-ring-expanded derivatives (**16**~**18**) were synthesized to further investigate the structure–activity relationships. 2-Aminobiphenyl (**14**) was acylated with chloroacetylchloride to give compound **15**. According to the literature [39], ring closure resulting in dibenzoazepinone (**16**) was achieved by an intramolecular Friedel–Crafts alkylation. Then compound **16** reacted with CH_3_I to form compound **17**, and compound **17** was reduced by LiAlH_4_ to give compound **18** with good yield.

### 2.2. Antiviral Activity and Structure−Activity Relationships (SARs)

The antiviral activities of the target compounds against TMV in three modes (inactivation effect, curative effect, and protection effect in vivo) with compound **2**, commercial antiviral agent ribavirin, and ningnanmycin as standards are shown in Table 1 and Table 2. Generally, the results showed that many compounds (**3f**, **3h**, **4c**, **4e**, **4i**, **5b**, **13b**, **13c**, **16**, and **18**) exhibited better anti-TMV activities (inactivation effect) than ribavirin at 500 mg/L. Interestingly, the anti-TMV activities of compounds **13b** and **18** were as good as those of ningnanmycin at 100 mg/L. In terms of the phenanthridin-6(5*H*)-one derivatives (**3a**~**i** and **13a**~**e**), compound **3h** with no substituents on either of the benzene rings exhibited good anti-TMV activities (inactivation effect) at 500 mg/L, while the other compounds (**3a**~**g** and **3i**), for which the benzene rings had at least one substituent, had decreased anti-TMV activities in various degrees. This result indicated that benzene ring electron-withdrawing substituents (F and Br) or an electron-donating substituent (OCH_3_) were not conducive to the improvement in the anti-TMV activities of phenanthridin-6(5*H*)-one derivatives. Although the anti-TMV activities (inactivation effect) of compounds **13a** and **13c**~**e** were lower than those of **3h**, the anti-TMV activity of **13b** was significantly increased, especially the protection effect; these results indicated that modification of the nitrogen atom of phenanthridone could enhance its anti-TMV activity. In terms of the phenanthridine derivatives (**4a~i**) and 5-methylphenanthridinium chloride derivatives (**5a~i**), the compounds bearing no substituents on the benzene ring (**4h** and **5h**) exhibited weak anti-TMV activities, while most of the compounds with substituents showed better anti-TMV activities than **4h** and **5h**, especially compounds **4c**, **4e**, **4i**, and **5b**. These results indicated that adding functional groups to benzene rings could improve the anti-TMV activities of the phenanthridine derivatives (**4a**~**i**) and 5-methylphenanthridinium chloride derivatives (**5a**~**i**). Compounds **16** and **17** were B-ring-expanded derivatives of **3h** and **13a**, respectively. The anti-TMV activities of compounds **16** and **17** were slightly better than those of compounds **3h** and **13a**. These results revealed that extending the size of the B-ring may not decrease their anti-TMV activities. Satisfyingly, the reductive product **18** showed excellent anti-TMV activity; in particular, the protection effect was near that of ningnanmycin.

### 2.3. Preliminary Mode of Action of Anti-TMV

**Figure 2:**
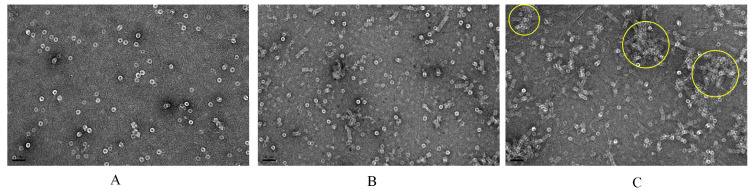
Results of in vitro 20S disk inhibition reaction under transmission electron microscope: (**A**) blank; (**B**) DMSO; and (**C**) compound **18** and the aggregates indicated by yellow circles.

**Figure 3:**
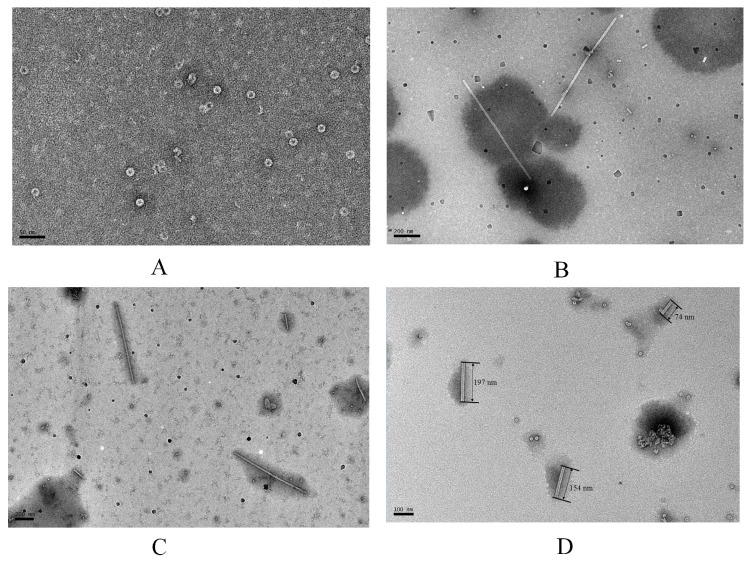
Results of in vitro TMV nanorod assembly reaction under transmission electron microscope: (**A**) 20S disk without RNA; (**B**) 20S disk with RNA; (**C**) 20S disk with RNA and DMSO; and (**D**) 20S disk with RNA and compound **18**.

The study of the mode of action of a potent molecule is very important and challenging in the development of pesticides [40]. It is known that the TMV capsid protein can turn into a 20S disk, following which the 20S disk and TMV RNA can further assemble to form rod-shaped TMV virus particles [41]. A preliminary study was undertaken to explore the effect of compound **18** which showed good anti-TMV activity on the TMV assembly. First, the effect of compound **18** on the TMV capsid protein was examined. In the control group (blank control, Figure 2A; DMSO control, Figure 2B), the 20S disks were well formed and were scattered around. When treated with compound **18** (Figure 2C), the 20S disks were also formed but were aggregated to form an irregular polymer (the part circled in yellow in Figure 2C). Subsequently, the effect of compound **18** on TMV self-assembly was explored. TMV cannot self-assemble without RNA (Figure 3A). The control test (Figure 3B,C) showed that TMV could self-assemble normally to form rod-shaped particles with a length of about 300 nm. When adding compound **18** (Figure 3D), the length of the rod-shaped particles was less than 300 nm (their average length was 142 nm), which indicated that the elongation of the assembly process was impeded. These findings suggest that compound 18 may interfere with the normal assembly process of TMV. However, it is important to note that this study is preliminary, and further experiments are required to fully understand the mode of action of compound **18**. Additional studies, including the examination of other potential targets and a comparison with other compounds, will be necessary to establish a more comprehensive understanding of its inhibitory mechanism.

### 2.4. Molecular Docking

**Figure 4:**
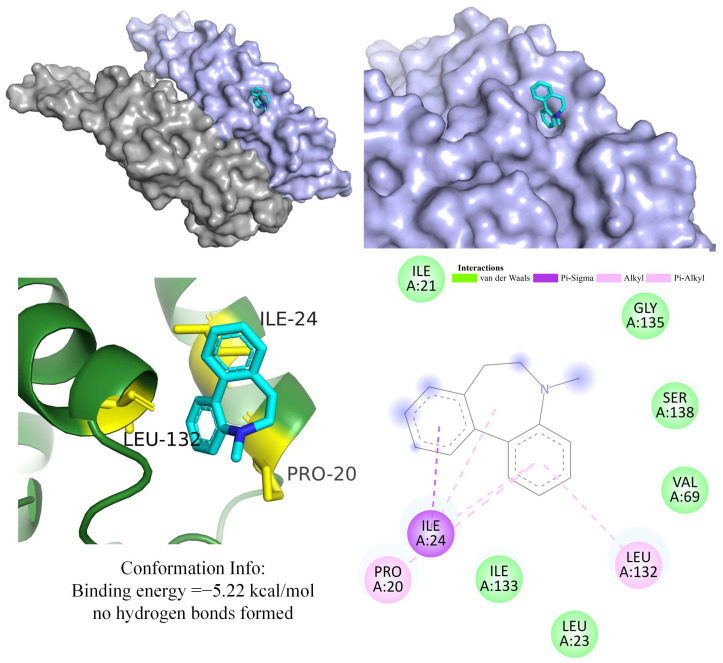
Molecular docking results of compound **18** with the TMV coat protein.

To further explore the possible mechanism of action, representative compound **18** and the TMV coat protein (PDB code: 1EI7) were chosen as the ligand and the receptor, respectively, for molecular docking studies using AutoDockTools. The experimental results are shown in Figure 4. The A and B chains of the TMV coat protein are represented in light blue and grey, respectively. The optimal binding mode of compound **18** to the TMV coat protein is on chain A, with a binding energy of −5.22 kcal/mol. Compound **18** primarily interacts with the protein through van der Waals forces with the amino acid residues ILE-21, LEU-23, VAL-69, ILE-133, GLY-135, and SER-138, π-σ interactions with the ILE-24 residue, alkyl interactions with the ILE-24 and π-alkyl interactions with the ILE-24, PRO-20 and LEU-132 residues. These interactions were relatively weak. Future structural optimizations might involve the addition of polar functional groups to enhance hydrogen bonding, which could consequently improve the activity of the compounds.

## 3. Materials and Methods

### 3.1. Chemicals

Reagents were purchased from commercial sources and used as received. All anhydrous solvents used for synthesis were dried and purified using standard techniques prior to use.

### 3.2. Instruments

The melting points of the synthesized compounds were tested using an X-4 binocular microscope (Beijing Tech Instruments Co., Beijing, China). NMR spectra were obtained using a Bruker AV 400 spectrometer (Bruker Corp., Fallanden, Switzerland) in CDCl_3_, DMSO-*d*_6_, or CD_3_OD solutions with tetramethylsilane (TMS) as the internal standard. The progress of the reaction was monitored by thin-layer chromatography on silica gel GF-254 and detected by UV. High-resolution mass spectra were obtained with an ESI-FTICR MS spectrometer (Ionspec, 7.0 T, Bruker, Saarbrucken, Germany). In vitro 20S disk inhibition reaction and in vitro TMV nanorod assembly reaction were tested via transmission electron microscopy (Tecnai G2 F20, TEI, Hillsboro, OR, USA).

### 3.3. Chemical Synthesis

Compounds **3a**~**g** [34], **3h** [35], **3i** [36], **5a**~**i** [37], **13a**~**e** [38], and **16**~**18** [39] were prepared according to the previously reported procedures with slight modification and the detailed synthetic method and data can be found in the Supporting Information. Compounds **4a**~**i** were prepared using our method.

Trisphaeridine (**4a**)

To a solution of **3a** (1.0 g, 4.2 mmol) in dry THF (50 mL), lithium aluminum hydride (LiAlH_4_, 0.26 g, 6.3 mmol) was added in several portions in an ice bath. Then, the reaction was refluxed for 4 h, quenched with water (1 mL), and filtered. The filtrate was concentrated under reduced pressure. The crude residue was dissolved in CH_2_Cl_2_ (100 mL) and then trifluoroacetic acid (CF_3_COOH, 1 mL) was added. After stirring at room temperature with air bubbling for 6 h, the mixture was then basified (pH = 8) with a saturated NaHCO_3_ solution and extracted with CH_2_Cl_2_ (30 mL × 3). The combined organic phase was washed with brine (30 mL), dried over anhydrous Na_2_SO_4_, and concentrated. The residue was purified using column chromatography on silica gel with petroleum ether/ethyl acetate (10:1, *v*/*v*) to give trisphaeridine (**4a**, 0.65 g, 69%) as a white solid; mp 141–142 °C (lit. [42] mp 140–142 °C); ^1^H NMR (400 MHz, CDCl_3_) *δ* 9.05 (s, 1H), 8.32 (d, *J* = 8.1 Hz, 1H), 8.13 (d, *J* = 8.1 Hz, 1H), 7.83 (s, 1H), 7.73–7.64 (m, 1H), 7.60 (dd, *J* = 11.1, 4.0 Hz, 1H), 7.27 (s, 1H), 6.12 (s, 2H).

Phenanthridine analogs **4b**~**i** were prepared similarly to **4a**.

2,4-Dimethoxyphenanthridine (**4b**)

White solid; mp 92–93 °C; yield 74%. ^1^H NMR (400 MHz, CDCl_3_) *δ* 9.43 (d, *J* = 8.7 Hz, 1H), 9.27 (s, 1H), 8.08 (t, *J* = 7.9 Hz, 1H), 7.92 (t, *J* = 7.8 Hz, 1H), 7.69 (t, *J* = 7.4 Hz, 1H), 7.47 (s, 1H), 6.85 (d, *J* = 2.4 Hz, 1H), 4.13 (s, 3H), 4.01 (s, 3H). ^13^C NMR (100 MHz, CDCl_3_) *δ* 159.8, 159.2, 154.4, 147.3, 132.8, 131.3, 128.7, 126.9, 125.9, 125.8, 109.7, 102.5, 99.6, 55.8, 55.6. HR-MS (ESI): Calcd for C_15_H_14_NO_2_ [M + H]^+^ 240.1019; found 240.1023.

8,9-Dimethoxyphenanthridine (**4c**)

Grey solid; mp 165–167 °C (lit. [42] mp 168–169 °C); yield 67%. ^1^H NMR (400 MHz, CDCl_3_) *δ* 9.20 (s, 1H), 8.45 (d, *J* = 7.8 Hz, 1H), 8.19 (d, *J* = 7.8 Hz, 1H), 7.88 (s, 1H), 7.71 (t, *J* = 6.9 Hz, 1H), 7.69–7.63 (m, 1H), 7.37 (s, 1H), 4.15 (s, 3H), 4.08 (s, 3H).

2-Fluoro-8,9-dimethoxyphenanthridine (**4d**)

Brown solid; mp 163–164 °C (lit. [43] mp 161–162 °C); yield 48%. ^1^H NMR (400 MHz, CDCl_3_) *δ* 9.10 (s, 1H), 8.13 (dd, *J* = 9.0, 5.7 Hz, 1H), 8.02 (dd, *J* = 10.2, 2.7 Hz, 1H), 7.73 (s, 1H), 7.46–7.39 (m, 1H), 7.36 (s, 1H), 4.14 (s, 3H), 4.08 (s, 3H).

2,9-Difluorophenanthridine (**4e**)

Yellow solid; mp 190–191 °C; yield 64%. ^1^H NMR (400 MHz, CDCl_3_) *δ* 9.21 (s, 1H), 8.21 (dd, *J* = 8.9, 5.6 Hz, 1H), 8.17–7.99 (m, 3H), 7.62–7.41 (m, 2H). ^13^C NMR (100 MHz, CDCl_3_) *δ* 164.3 (d, *J*_F–C_ = 253.7 Hz), 161.3 (d, *J*_F–C_ = 248.5 Hz), 151.6, 140.9, 134.3 (dd, *J*_F–C_ = 10.0, 4.1 Hz), 132.2 (d, *J*_F–C_ = 9.5 Hz), 131.8 (d, *J*_F–C_ = 9.8 Hz), 125.0 (dd, *J*_F–C_ = 9.0, 4.1 Hz), 123.2, 118.5 (d, *J*_F–C_ = 24.2 Hz), 117.6 (d, *J*_F–C_ = 24.2 Hz), 107.6 (d, *J*_F–C_ = 10.0 Hz), 107.3 (d, *J*_F–C_ = 10.7 Hz). HR-MS (ESI): Calcd for C_13_H_8_F_2_N [M + H]^+^ 216.0619; found 216.0624.

9-Fluoro-2,4-dimethoxyphenanthridine (**4f**)

Yellow solid; mp 259–260 °C; yield 80%. ^1^H NMR (400 MHz, CDCl_3_) δ 9.31 (s, 1H), 9.09 (dd, *J* = 12.6, 2.4 Hz, 1H), 8.17 (dd, *J* = 8.8, 6.1 Hz, 1H), 7.54 (d, *J* = 2.3 Hz, 1H), 7.49–7.42 (m, 1H), 6.86 (d, *J* = 2.4 Hz, 1H). ^13^C NMR (100 MHz, CDCl_3_) *δ* 165.7 (d, *J*_F–C_ = 254.9 Hz), 161.5, 159.2, 150.9, 142.9, 136.1 (d, *J*_F–C_ = 12.2 Hz), 132.8 (d, *J*_F–C_ = 10.2 Hz), 121.7, 116.4 (d, *J*_F–C_ = 25.4 Hz), 112.6 (d, *J*_F–C_ = 25.8 Hz), 109.9 (d, *J*_F–C_ = 4.4 Hz), 100.9, 99.5, 56.2, 56.1. HR-MS (ESI): Calcd for C_15_H_13_FNO_2_ [M + H]^+^ 258.0925; found 258.0930.

9-Fluorophenanthridine (**4g**)

White solid; mp 105–106 °C (lit. [42] mp 106–108 °C); yield 45%. ^1^H NMR (400 MHz, CDCl_3_) δ 9.25 (s, 1H), 8.46 (dd, *J* = 8.1, 1.1 Hz, 1H), 8.27–8.18 (m, 2H), 8.09 (dd, *J* = 8.8, 5.7 Hz, 1H), 7.84–7.76 (m, 1H), 7.75–7.68 (m, 1H), 7.46 (dt, *J* = 8.5, 2.4 Hz, 1H).

Phenanthridine (**4h**)

White solid; mp 98–99 °C (lit. [42] mp 100–101 °C); yield 82%. ^1^H NMR (400 MHz, CDCl_3_) *δ* 9.30 (s, 1H), 8.68–8.56 (m, 2H), 8.24–8.16 (m, 1H), 8.06 (d, *J* = 7.8 Hz, 1H), 7.92–7.83 (m, 1H), 7.80–7.65 (m, 3H).

2-Bromophenanthridine (**4i**)

Yellow solid; mp 160–161 °C (lit. [44] mp 162–163 °C); yield 67%. ^1^H NMR (400 MHz, CDCl_3_) δ 9.25 (s, 1H), 8.66 (d, *J* = 2.1 Hz, 1H), 8.49 (d, *J* = 8.3 Hz, 1H), 8.08–8.04 (m, 1H), 8.04–8.00 (m, 1H), 7.87 (ddd, *J* = 8.3, 7.2, 1.3 Hz, 1H), 7.80 (dd, *J* = 8.7, 2.1 Hz, 1H), 7.77–7.71 (m, 1H).

### 3.4. Biological Assay

The activities of the target compounds were tested on representative test organisms. To ensure the reliability of data, each bioassay was replicated at least three times. The antiviral activity against TMV was carried out using previously reported methods [14] with tobacco (*Nicotiana tabaccum* var. *Xanthinc*) as the test plant.

The detailed biological assay methods are also given in the Supporting Information.

### 3.5. Mode of Action of Anti-TMV Studies [45]

TMV was purified according to the method provided by Leberman [46]. The TMV capsid protein (TMV CP) was purified with the acetic acid method [47]. TMV RNA was purified using the method provided by Zimmern [48]. All the detailed methods are also given in the Supporting Information.

#### 3.5.1. In Vitro 20S Disk Inhibition Reaction

For drug tests, in vitro 20S disk inhibition reaction was performed at 20 °C for 12 h after adding 9.8 μL TMV capsid proteins (20 mg/mL) and 0.2 μL DMSO or drug (10 nmol/mL, 0.2 μL in DMSO). After the treatment, the sample was used for transmission electron microscope (TEM) characterization.

#### 3.5.2. In Vitro TMV Nanorod Assembly Reaction

Before the assembly of TMV nanorods, the 20S disk was prepared by incubating TMV capsid proteins (20 mg/mL) in 0.1 M phosphate buffer (pH 7.0) at 20 °C for 12 h. The TMV nanorod assembly reaction was performed by mixing 5 μL phosphate buffer (0.1 mol/L, pH 7.0), 4 μL 20S disk (20 mg/mL), and 1 μL TMV RNA (200 ng/μL). The assembly reaction was incubated at 20 °C for 12 h. For drug tests, in vitro TMV reconstitution inhibition reactions were performed by adding 4.8 μL phosphate buffer (0.1 mol/L, pH 7.0), 4 μL 20S disk (20 mg/mL), 1 μL TMV RNA (200 mg/mL), and 0.2 μL DMSO or drug (10 nmol/mL, 0.2 μL in DMSO). After the treatment, the sample was used for transmission electron microscope (TEM) characterization.

#### 3.5.3. Molecular Docking [49,50]

The structure of the TMV coat protein was downloaded from the RCSB Protein Data Bank (PDB ID: 1EI7) [51]. The molecular file for compound 18 (CID: 13095226) was downloaded from PubChem. The potential binding sites on the TMV coat protein were identified using AutoDockTools (version 1.5.7, The Scripps Research Institute, La Jolla, CA, USA), following Olson’s protocol [52]. The docking simulations were performed using the AutoDock algorithm. The global docking was conducted with a grid size that encompassed the entire protein. All other docking parameters are default values, with the following exceptions: ‘Number of GA Runs’ was set to 100, ‘Maximum Number of evals’ was set to 10,000,000, and ‘Maximum Number of generations’ was set to 100,000. The docking results were visualized using PyMOL (version 3.1.3, Schrödinger, New York, NY, USA) and the 2D ligand interaction diagrams were generated using Discovery Studio Visualizer (version 24.1.0, Build 23298, Dassault Systèmes, Vélizy-Villacoublay, France).

## 4. Conclusions

In summary, 35 compounds including crinasiadine (**3a**), trisphaeridine (**4a**), bicolorine (**5a**), and their derivatives were synthesized, and their anti-TMV activities were investigated for the first time. Many compounds (**3f**, **3h**, **4c**, **4e**, **4i**, **5b**, **13b**, and **13c**) exhibited better anti-TMV activities than ribavirin; in particular, compounds **13b** and **18** were as effective as ningnanmycin. The preliminary results suggested that compound **18** could impede the elongation of the TMV assembly process. In this study, phenanthridine analogs exhibit strong anti-TMV activities, highlighting their potential for crop protection. It is important to note that the capacity of phenanthrene compounds to intercalate with DNA and RNA raises substantial concerns regarding their potential toxicity to non-target organisms and their environmental impact [17]. While certain phenanthridine derivatives have been used safely in the treatment of livestock [21,22,23], the wider ecological implications of employing these compounds in crop protection demand further study to guarantee environmental safety. And it is believed that the phenanthridine structure is a promising small molecule, which could play an important role in crop protection.

## Figures and Tables

**Table 1:** In vivo antiviral activities (inactivation effect) of phenanthridin-6(5*H*)-one analogs (**3a**~**i**, **13a**~**e**, and **16**~**18**), phenanthridine analogs (**4a**~**i**), 5-methylphenanthridinium chloride analogs (**5a**~**i**), compound **2**, ribavirin, and ningnanmycin (NN) against TMV at 500 mg/L.

Sample	Inhibition Rate (%) *^a^*	Sample	Inhibition Rate (%) *^a^*	Sample	Inhibition Rate (%) *^a^*	Sample	Inhibition Rate (%) *^a^*
**3a**	19.5 ± 1.7	**4b**	25.3 ± 0.6	**5c**	37.2 ± 2.6	**13d**	37.8 ± 1.1
**3b**	31.8 ± 2.4	**4c**	40.9 ± 1.1	**5d**	19.2 ± 4.5	**13e**	31.6 ± 1.1
**3c**	33.7 ± 1.8	**4d**	34.9 ± 3.5	**5e**	31.1 ± 0.2	**16**	41.2 ± 3.5
**3d**	31.9 ± 3.3	**4e**	43.0 ± 2.5	**5f**	36.8 ± 0.8	**17**	32.5 ± 1.9
**3e**	29.2 ± 1.1	**4f**	30.9 ± 4.8	**5g**	38.4 ± 0.3	**18**	49.2 ± 1.6
**3f**	41.3 ± 0.2	**4g**	33.5 ± 1.4	**5h**	26.3 ± 2.1	**2**	44.7 ± 0.5
**3g**	35.1 ± 2.4	**4h**	21.5 ± 3.4	**5i**	35.2 ± 1.1	**ribavirin**	39.5 ± 0.3
**3h**	46.8 ± 0.2	**4i**	45.9 ± 4.3	**13a**	27.0 ± 4.0	**NN**	58.8 ± 2.4
**3i**	37.5 ± 1.0	**5a**	34.0 ± 4.4	**13b**	59.4 ± 1.8		
**4a**	35.2 ± 2.6	**5b**	49.7 ± 0.8	**13c**	42.9 ± 1.9		

*^a^* The effect (inhibition rate) is presented as the mean ± SD (%). The deep blue color emphasizes an inhibition rate greater than 39.5% (ribavirin).

**Table 2:** In vivo antiviral activities of phenanthridin-6(5*H*)-one analogs (**3a**~**i**, **13a**~**e**, and **16**~**18**), phenanthridine analogs (**4a**~**i**), 5-methylphenanthridinium chloride analogs (**5a**~**i**), compound **2**, ribavirin, and ningnanmycin (NN) against TMV.

Sample	Conc. (mg/L)	Inhibition Rate (%) *^a^*			Sample	Conc. (mg/L)	Inhibition Rate (%)		
		Inactivation Effect	Curative Effect	Protection Effect			Inactivation Effect	Curative Effect	Protection Effect
**3f**	500	41.3 ± 0.2	46.9 ± 1.8	38.1 ± 2.3	**13c**	500	42.9 ± 1.9	35.4 ± 3.6	44.7 ± 1.1
	100	5.8 ± 0.5	11.5 ± 2.7	7.4 ± 0.9		100	7.2 ± 0.7	6.0 ± 0.4	13.5 ± 0.2
**3h**	500	46.8 ± 0.2	42.1 ± 1.4	41.7 ± 0.6	**16**	500	41.2 ± 3.5	44.4 ± 2.0	37.9 ± 1.2
	100	9.3 ± 0.7	12.5 ± 2.0	0		100	5.3 ± 1.0	8.6 ± 0.6	0
**4c**	500	40.9 ± 1.1	36.7 ± 1.3	47.0 ± 3.8	**18**	500	49.2 ± 1.6	47.3 ± 0.2	54.4 ± 2.5
	100	15.1 ± 0.6	9.6 ± 1.8	17.9 ± 2.0		100	16.7 ± 1.1	19.2 ± 0.8	22.0 ± 2.1
**4e**	500	43.0 ± 2.5	40.1 ± 3.4	35.6 ± 4.5	**2**	500	44.7 ± 0.5	40.9 ± 3.3	39.1 ± 2.9
	100	9.8 ± 0.9	11.0 ± 0.7	7.8 ± 1.3		100	6.6 ± 0.1	0	0
**4i**	500	45.9 ± 4.3	41.7 ± 2.0	47.8 ± 0.6	**ribavirin**	500	39.5 ± 0.3	37.2 ± 1.4	40.2 ± 0.8
	100	12.1 ± 0.7	18.6 ± 3.5	9.1 ± 1.0		100	11.3 ± 0.9	13.6 ± 0.8	9.5 ± 1.2
**5b**	500	49.7 ± 0.8	47.1 ± 2.9	51.3 ± 3.7	**NN**	500	58.8 ± 2.4	55.9 ± 0.7	57.1 ± 0.6
	100	17.6 ± 1.4	9.4 ± 2.5	15.2 ± 0.5		100	26.4 ± 0.9	24.0 ± 1.2	27.2 ± 1.5
**13b**	500	59.4 ± 1.8	55.6 ± 1.0	52.3 ± 2.5					
	100	15.8 ± 3.6	18.0 ± 0.4	21.5 ± 2.0					

***^a^*** The effect (inhibition rate) is presented as mean ± SD (%).

## Data Availability

Data have been stored in the library repository of Nankai University, Tianjin, China.

## Supplementary Materials

The supporting information can be downloaded at https://www.mdpi.com/article/10.3390/ijms26031103/s1. References [14,34,35,36,37,38,39,42,46,47,53,54,55,56,57,58] are cited in the supplementary materials.
