# Supplementary material for: Discovery of Crinasiadine, Trisphaeridine, Bicolorine, and Their Derivatives as Anti-Tobacco Mosaic Virus (TMV) Agents
Source: Int J Mol Sci. 2025 Jan 27;26(3):1103. doi: 10.3390/ijms26031103 (PMC11816930; doi:10.3390/ijms26031103)
Supplement: Supplementary file 1 [file ijms-26-01103-s001.zip › ijms-3377441-supplementary.pdf]

Discovery of Crinasiadine, Trisphaeridine, Bicolorine, and Their Derivatives as Anti-Tobacco Mosaic Virus (TMV) Agents

(Supporting Information)

Tables of Content

1.The detailed synthetic method and data of compounds **3a~g**, **3h**, **3i**, **5a~i**, **13a~e**, and **16~18**..2

2. The detailed biological assay methods 14

3. Mode of action of anti-TMV studies 15

4. ^{1}H NMR and ^{13}C NMR spectra for new compounds. 18

1.The detailed synthetic method and data of compounds **3a~g**[1], **3h**[2], **3i**[3], **5a~i**[4], **13a~e**[5], and **16~18**[6]

Synthesis of phenanthridin-6(5*H*)-one analogues **3a~g**

[1,3]Dioxolo[4,5-j]phenanthridin-6(5*H*)-one (**3a**)

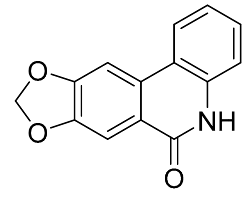

3a

To a solution of 6-bromo-1,3-benzodioxole-5-carboxylic acid (**10a**, 2.45 g, 10 mmol) in anhydrous toluene (25 mL) was added dropwise $SOCl_2$ (1.5 mL, 20 mmol). The mixture was stirred at room temperature for 5 min and then refluxed for 8 h. The mixture was cooled to room temperature and concentrated in vacuo to give the corresponding 6-bromo-1,3-benzodioxole-5-carbonyl chloride.

The mixture of aniline (1.4 mL, 15 mmol) and trimethylamine (Et_3N, 2 mL, 15 mmol) was added dropwise to a solution of the crude 6-bromo-1,3-benzodioxole-5-carbonyl chloride in anhydrous dichloromethane (CH_2Cl_2, 50 mL) at 0 °C. After completion of the addition, the mixture was stirred at room temperature for 5 h, then it was poured into 50 mL of water and acidified (pH = 6) with dilute hydrochloric acid. Then extra 50 mL of CH_2Cl_2 was added and the organic phase was washed with water (30 mL×3) in a separating funnel. Subsequently, the organic phase was basified (pH = 8) with saturated aqueous sodium bicarbonate ($NaHCO_3$) solution, and extracted with CH_2Cl_2 (30 mL×3). The organic layer was combined, washed with brine, dried over anhydrous sodium sulfate (Na_2SO_4), filtered, and concentrated. The crude residue was

dried to give compound **11a** as a yellow solid (2.6 g, 89% for two steps).

Compound **11a** (320 mg, 1 mmol), potassium *t*-butoxide (*t*-BuOK, 560 mg, 5 mmol) and azodiisobutyronitrile (AIBN, 33 mg, 0.2 mmol) were added into benzene (6 mL) in a sealed tube. The mixture was heated at 110 °C for 12 h under argon. After this, the chilled mixture was acidified (pH = 6) with saturated ammonium chloride (NH_4Cl) solution. Then the mixture was filtered and the filter cake was washed with water and CH_2Cl_2. The filter cake was dried to give [1,3]dioxolo[4,5-j]phenanthridin-6(5*H*)-one (**3a**, 172 mg, 72%) as a gray solid; mp > 300 ºC (lit.[7] mp 365–367 °C). ^{1}H NMR (400 MHz, DMSO-d_6) δ 11.63 (s, 1H), 8.30 (d, *J* = 7.8 Hz, 1H), 8.05 (s, 1H), 7.64 (s, 1H), 7.43 (t, *J* = 7.2 Hz, 1H), 7.33 (d, *J* = 8.3 Hz, 1H), 7.22 (t, *J* = 7.3 Hz, 1H), 6.23 (s, 2H)

Compounds **3b~g** were prepared like **3a**.

2,4-Dimethoxyphenanthridin-6(5*H*)-one (**3b**)

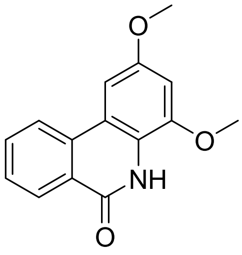

3b

Yellow solid; m.p. 234-235 °C; yield 65%. ^{1}H NMR (400 MHz, DMSO-d_6) δ 11.58 (s, 1H), 9.04 (d, *J* = 8.4 Hz, 1H), 8.31 (d, *J* = 7.6 Hz, 1H), 7.76 (t, *J* = 7.6 Hz, 1H), 7.52 (t, *J* = 7.5 Hz, 1H), 6.60 (s, 1H), 6.51 (s, 1H), 4.02 (s, 3H), 3.83 (s, 3H).

8,9-Dimethoxyphenanthridin-6(5*H*)-one (**3c**)

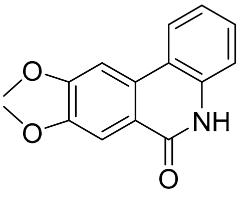

3c

Yellow solid; mp >300 ºC (lit.[1] mp 314–316 °C); yield 70%. ^{1}H NMR (400 MHz, DMSO-d_6) δ 11.61 (s, 1H), 8.40 (d, *J* = 7.8 Hz, 1H), 7.89 (s, 1H), 7.71 (s, 1H), 7.43 (d, *J* = 7.6 Hz,

1H), 7.35 (t, *J* = 9.5 Hz, 1H), 7.26 (d, *J* = 7.5 Hz, 1H), 4.02 (s, 3H), 3.91 (s, 3H).

2-Fluoro-8,9-dimethoxyphenanthridin-6(5*H*)-one (**3d**)

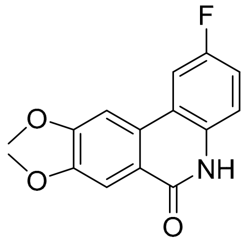

3d

Yellow solid; mp 253–254 °C; yield 50%. ^{1}H NMR (400 MHz, DMSO-d_6) δ 11.66 (s, 1H), 8.33 (d, *J* = 9.9 Hz, 1H), 7.89 (s, 1H), 7.71 (s, 1H), 7.44 – 7.26 (m, 2H), 4.02 (s, 3H), 3.91 (s, 3H). ^{13}C NMR (100 MHz, DMSO-d_6) δ 160.6, 158.3 (d, $J_{F\text{–}C}$ = 237.7 Hz), 153.7, 150.3, 133.1, 128.8 (d, $J_{F\text{–}C}$ = 3.2 Hz), 120.0, 119.4 (d, $J_{F\text{–}C}$ = 8.6 Hz), 117.9 (d, $J_{F\text{–}C}$ = 8.1 Hz), 116.5(d, $J_{F\text{–}C}$ = 23.9 Hz), 109.5 (d, $J_{F\text{–}C}$ = 24.5 Hz), 108.2, 105.1, 56.6, 56.0. HR-MS (ESI):Calcd for $C_{15}H_{13}FNO_3$ [M + H]$^+$ 274.0874; found 274.0875.

2,9-Difluorophenanthridin-6(5*H*)-one (**3e**)

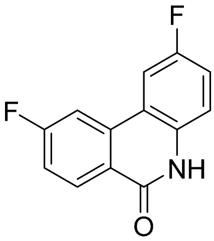

3e

White solid; mp 265-266 °C; yield 50%. ^{1}H NMR (400 MHz, DMSO-d_6) δ 11.78 (s, 1H), 8.44 – 8.34 (m, 2H), 8.30 (dd, *J* = 10.3, 1.8 Hz, 1H), 7.52 (dt, *J* = 8.6, 2.0 Hz, 1H), 7.46 – 7.34 (m, 2H).

9-Fluoro-2,4-dimethoxyphenanthridin-6(5*H*)-one (**3f**)

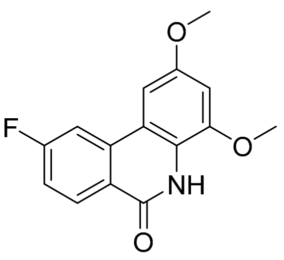

3f

Yellow solid; mp 254-255 °C; yield 72%. ^{1}H NMR (400 MHz, DMSO-d_6) δ 11.62 (s, 1H), 8.70 (d, *J* = 13.0 Hz, 1H), 8.35 (t, *J* = 7.7 Hz, 1H), 7.37 (t, *J* = 7.0 Hz, 1H), 6.59 (s, 1H), 6.51 (s, 1H), 4.03 (s, 3H), 3.83 (s, 3H). ^{13}C NMR (100 MHz, DMSO-d_6) δ 165.0 (d, $J_{F\text{–}C}$ = 246.7 Hz), 161.3, 160.7, 160.0, 140.5, 137.0

(d, $J_{F\text{–}C}$ = 11.4 Hz), 130.7 (d, $J_{F\text{–}C}$ = 10.3 Hz), 121.7, 114.2 (d, $J_{F\text{–}C}$ = 23.1 Hz), 112.4 (d, $J_{F\text{–}C}$ = 25.9 Hz), 101.0 (d, $J_{F\text{–}C}$ = 2.8 Hz), 94.3, 92.8, 56.5, 55.8. HR-MS (ESI):Calcd for $C_{15}H_{13}FNO_3$ $[M + H]^+$ 274.0874； found 274.0873.

9-Fluorophenanthridin-6(5*H*)-one (**3g**)

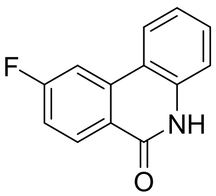

3g

Gray solid; mp > 300 ºC (lit.[1] mp 302–304 °C); yield 53%.. ^{1}H NMR (400 MHz, DMSO-d_6) δ 11.75 (s, 1H), 8.49 – 8.28 (m, 3H), 7.60 – 7.44 (m, 1H), 7.42 – 7.33 (m, 1H), 7.27 (t, *J* = 7.5 Hz, 1H).

Synthesis of phenanthridin-6(5*H*)-one (**3h**)

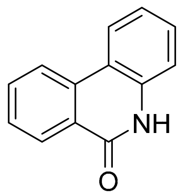

3h

To a solution of 9-fluorenone (**12**, 3.6 g, 20 mmol) in the methanol (50 mL) was added hydroxylamine hydrochloride ($NH_2OH·HCl$, 1.7 g, 24 mmol) and sodium acetate (CH_3CO_2Na, 3.2 g, 24 mmol), and the mixture was stirred at room temperature for 6 h. The solvent was evaporated, and the remaining solid was dissolved in ethyl acetate (100 mL) and water (50 mL), then the organic phase was washed with water (30 mL×3) in a separating funnel, dried over anhydrous Na_2SO_4, filtered, and concentrated. The crude residue was dried to give 9-fluorenone oxime as a yellow solid (3.8 g, 97%); mp 207–208 °C (lit.[2] mp 207–209 °C). ^{1}H NMR (400 MHz, DMSO-d_6) δ 12.54 (s, 1H), 8.34 (d, *J* = 7.6 Hz, 1H), 7.89 (d, *J* = 7.5 Hz, 1H), 7.85 (d, *J* = 7.5 Hz, 1H), 7.70 (d, *J* = 7.5 Hz, 1H), 7.51 (t, *J* = 7.3 Hz, 1H), 7.48–7.30 (m, 3H). 9-Fluorenone oxime (1.85g, 10mmol) was heated with stirring in polyphosphoric acids (PPA, 12 g) at 150 °C for 1 h, then the mixture was

maintained at room temperature without stirring for 15 h. The cool mixture was then warmed slightly so the viscous acidic mixture could be poured over 3 inches of ice in a 50 mL beaker. After stirring several minutes, a pale tan precipitate was liberated. The precipitate was washed several times with water and collected by vacuum filtration. The filter cake was dried to give phenanthridin-6(5*H*)-one (**3h**, 1.9 g, 97%); mp 289–290 °C (lit.[2] mp 290–292 °C). ^{1}H NMR (400 MHz, DMSO-d_6) δ 11.71 (s, 1H), 8.52 (d, *J* = 8.1 Hz, 1H), 8.40 (d, *J* = 8.0 Hz, 1H), 8.33 (d, *J* = 7.4 Hz, 1H), 7.87 (t, *J* = 7.1 Hz, 1H), 7.65 (t, *J* = 7.5 Hz, 1H), 7.50 (t, *J* = 7.3 Hz, 1H), 7.37 (d, *J* = 7.8 Hz, 1H), 7.27 (t, *J* = 7.5 Hz, 1H).

Synthesis of 2-bromophenanthridin-6(5*H*)-one (**3i**)

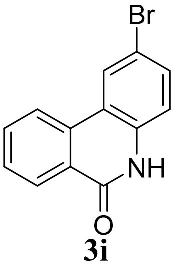

3i

To a solution of phenanthridin-6(5*H*)-one (1 g, 5.1 mmol) in dimethylformamide (DMF, 25 mL) at 60 °C, *N*-bromosuccinimide (1 g, 5.6 mmol) was added in several portions. The mixture was stirred at room temperature for 24 h. Then the mixture was poured into water, and a gray precipitate was liberated. The precipitate was washed several times with water and collected by vacuum filtration. The filter cake was dried to give 2-bromophenanthridin-6(5*H*)-one (**3i**, 1.15 g, 80%), mp >300 ºC (lit.[3] mp 323–324 °C). ^{1}H NMR (400 MHz, DMSO-d_6) δ 11.82 (s, 1H), 8.61 (d, *J* = 2.0 Hz, 1H), 8.58 (d, *J* = 8.1 Hz, 1H), 8.32 (dd, *J* = 7.9, 1.0 Hz, 1H), 7.91–7.82 (m, 1H), 7.70 (d, *J* = 7.4 Hz, 1H), 7.67–7.63 (m, 1H), 7.32 (d, *J* = 8.7 Hz, 1H).

Synthesis of 5-methyl-[1,3]dioxolo[4,5-j]phenanthridinium chloride (**5a**)

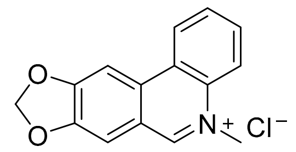

5a

Dimethyl sulfate ($(CH_3)_2SO_4$, 0.4 mL, 4.2 mmol) was added to a suspension of **4a** (0.2 g, 1 mmol) in nitrobenzene (4 mL) and xylene (2 mL). Within 30 min, the mixture was heated to 140 °C and stirred for 1.5 h. The chilled mixture was poured into diethyl ether (Et_2O, 25 mL). The precipitate was allowed to settle down and filtered off, washed with Et_2O (5 mL×3). The dry filter cake was dissolved in water (40 mL), then hydrochloric acid (0.5 mL) was added, and the mixture was refluxed for 0.5 h. Barium chloride ($BaCl_2$, 0.2 g, 1 mmol) was added into the chilled mixture, the precipitate was filtered off, and the filtrate was concentrated by vacuum. The residue was recrystallized from methanol to give 5-methyl-[1,3]dioxolo[4,5-j]phenanthridinium chloride as a yellow solid (**5a**, 0.22 g, 79%); mp 250–251 °C (lit.[8] mp 252–254 °C). ^1H NMR (400 MHz, CD_3OD) δ 9.71 (s, 1H), 8.94 (d, J = 8.3 Hz, 1H), 8.45 (s, 1H), 8.42 (d, J = 8.7 Hz, 1H), 8.10 (t, J = 7.9 Hz, 1H), 8.03 (t, J = 7.7 Hz, 1H), 7.77 (s, 1H), 6.43 (s, 2H), 4.64 (s, 3H).

5-Methylphenanthridinium chloride analogues **5b~i** were prepared like **5a**

2,4-Dimethoxy-5-methylphenanthridinium chloride（**5b**）

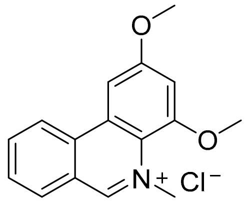

5b

Yellow solid; mp 217–218 °C; yield 70%. ^1H NMR (400 MHz, CD_3OD) δ 9.97 (s, 1H), 9.60 (d, J = 8.8 Hz, 1H), 8.45 (d, J = 8.1 Hz, 1H), 8.26 (ddd, J = 8.7, 7.1, 1.4 Hz, 1H), 7.93 (t, J = 7.2 Hz, 1H), 7.41 (d, J = 2.2 Hz, 1H), 7.27 (d, J = 2.2 Hz, 1H), 4.65 (s, 3H), 4.25 (s, 3H), 4.13 (s, 3H). ^{13}C NMR (100 MHz, CD_3OD) δ

162.8, 160.4, 155.2, 137.8, 137.5, 134.7, 132.3, 128.0, 126.6, 122.8, 111.1, 101.4, 93.3, 56.0, 55.8, 46.0. HR-MS (ESI): Calcd for $C_{16}H16NO_2^+$ [M - Cl]$^+$ 254.1176；found 254.1178.

8,9-Dimethoxy-5-methylphenanthridinium chloride（**5c**）

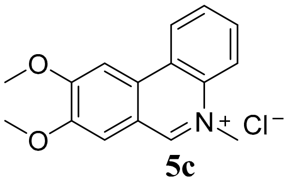

5c

Yellow solid; m.p. 204–205 °C; yield 80%. ^{1}H NMR (400 MHz, CD_3OD) δ 9.72 (s, 1H), 9.05 (d, J = 8.0 Hz, 1H), 8.42 (d, J = 8.4 Hz, 1H), 8.33 (s, 1H), 8.13 – 8.00 (m, 2H), 7.86 (s, 1H), 4.65 (s, 3H), 4.25 (s, 3H), 4.09 (s, 3H).

2-Fluoro-8,9-dimethoxy-5-methylphenanthridinium chloride (**5d**)

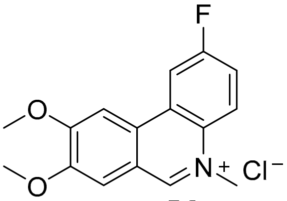

5d

Yellow solid; mp 220–221 °C; yield 73%. ^{1}H NMR (400 MHz, CD_3OD) δ 9.72 (s, 1H), 8.86 (dd, J = 9.5, 2.0 Hz, 1H), 8.50 (dd, J = 9.8, 4.5 Hz, 1H), 8.31 (s, 1H), 7.95 – 7.83 (m, 2H), 4.66 (s, 3H), 4.25 (s, 3H), 4.10 (s, 3H).

2,9-Difluoro-5-methylphenanthridinium chloride（**5e**）

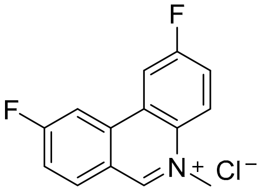

5e

Yellow solid; m.p. 234–235 °C; yield 59%. ^{1}H NMR (400 MHz, CD_3OD) δ 10.07 (s, 1H), 8.88 – 8.79 (m, 2H), 8.68 (dd, J = 8.9, 5.5 Hz, 1H), 8.63 (dd, J = 9.5, 4.6 Hz, 1H), 8.05 – 7.97 (m, 1H), 7.94 (td, J = 8.6, 2.2 Hz, 1H), 4.74 (s, 3H). ^{13}C NMR (100 MHz, CD_3OD) δ 168.4 (d, J = 262.3 Hz), 162.8 (d, J = 252.9 Hz), 154.4, 137.7 (dd, J_{F-C} = 11.9, 4.0 Hz), 136.5 (d, J_{F-C} = 11.4 Hz), 131.4 (d, J_{F-C} = 1.2 Hz), 127.8 (dd, J_{F-C} = 10.0, 4.5 Hz), 122.7 (d, J_{F-C} = 9.7 Hz), 121.3 (d, J_{F-C} = 1.0 Hz), 121.2 (d, J_{F-C} = 25.3 Hz),

120.3 (d, J_{F-C} = 25.1 Hz), 110.3 (d, J_{F-C} = 25.1 Hz), 109.4 (d, J_{F-C} = 24.9 Hz), 45.6. HR-MS (ESI): Calcd for $C_{14}H_{10}F_2N^+$ [M - Cl]$^+$ 230.0776；found 230.0778.

9-Fluoro-2,4-dimethoxy-5-methylphenanthridinium chloride (**5f**)

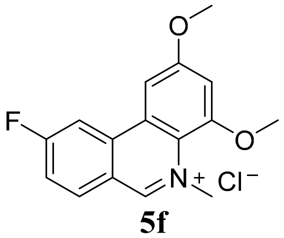

5f

Yellow solid; mp 206–207 °C; yield 75%. ^{1}H NMR (400 MHz, CD_3OD) δ 9.99 (s, 1H), 9.26 (dd, J = 12.7, 2.4 Hz, 1H), 8.55 (dd, J = 9.0, 6.1 Hz, 1H), 7.77 (ddd, J = 8.9, 7.9, 2.4 Hz, 1H), 7.42 (d, J = 2.2 Hz, 1H), 7.30 (d, J = 2.2 Hz, 1H), 4.64 (s, 3H), 4.27 (s, 3H), 4.14 (s, 3H). ^{13}C NMR (100 MHz, CD_3OD) δ 168.0 (d, J = 259.5 Hz), 163.4, 160.4, 154.6, 137.6, 137.1 (d, J = 13.4 Hz), 136.2 (d, J = 11.8 Hz), 120.0, 117.9 (d, J = 25.5 Hz), 112.0 (d, J = 27.3 Hz), 110.5 (d, J_{F-C} = 4.4 Hz), 101.5 (s), 93.7 (s), 56.3, 56.1, 46.2. HR-MS (ESI): Calcd for $C_{16}H_{15}FNO_2^+$ [M - Cl]$^+$ 272.1081；found 272.1084.

9-Fluoro-5-Methylphenanthridinium chloride (**5g**)

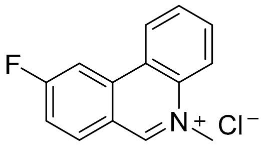

5g

Yellow solid, mp 199–200 °C; yield 75%. ^{1}H NMR (400 MHz, CD_3OD) δ 10.11 (s, 1H), 9.07 (dd, J = 8.3, 1.1 Hz, 1H), 8.84 (dd, J = 10.3, 2.2 Hz, 1H), 8.67 (dd, J = 8.9, 5.6 Hz, 1H), 8.55 (d, J = 8.6 Hz, 1H), 8.27 – 8.18 (m, 1H), 8.14 (dd, J = 11.3, 4.1 Hz, 1H), 7.91 (td, J = 8.7, 2.3 Hz, 1H), 4.74 (s, 3H). ^{13}C NMR (100 MHz, CD_3OD) δ 168.4 (d, J_{F-C} = 262.0 Hz), 154.8, 138.2 (d, J_{F-C} = 11.9 Hz), 136.5 (d, J_{F-C} = 11.4 Hz), 134.5, 132.7, 130.4 (s), 125.4 (d, J_{F-C} = 4.5 Hz), 121.0, 119.8 (d, J_{F-C} = 25.3 Hz), 119.5, 108.8 (d, J_{F-C} = 24.7 Hz), 45.3. HR-MS (ESI):Calcd for $C_{14}H_{11}FN^+$ [M - Cl]$^+$ 212.0870；found 212.0872.

5-Methylphenanthridinium chloride (**5h**)

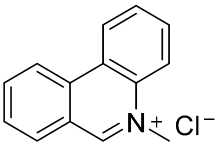

5h

Yellow solid; m.p. 168–169 °C (lit.[9] mp 169–170 °C); yield 79%. ^{1}H NMR (400 MHz, CD_3OD) δ 10.12 (s, 1H), 9.14 (dd, J = 8.1, 1.5 Hz, 1H), 9.09 (d, J = 8.4 Hz, 1H), 8.59 – 8.51 (m, 1H), 8.46 – 8.36 (m, 1H), 8.21 – 8.08 (m, 3H), 4.76 (s, 3H).

2-Bromo-5-methylphenanthridinium chloride (**5i**)

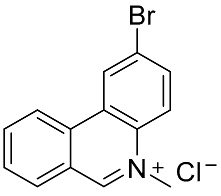

5i

Yellow solid; mp 243–244 °C; yield 73%. ^{1}H NMR (400 MHz, CD_3OD) δ 10.12 (s, 1H), 9.32 (d, J = 2.0 Hz, 1H), 9.06 (d, J = 8.4 Hz, 1H), 8.58 (d, J = 8.0 Hz, 1H), 8.46 (d, J = 9.2 Hz, 1H), 8.43 – 8.38 (m, 1H), 8.28 (dd, J = 9.2, 2.1 Hz, 1H), 8.13 (t, J = 7.6 Hz, 1H), 4.74 (s, 3H). ^{13}C NMR (100 MHz, CD_3OD) δ 157.5, 139.7, 136.3, 135.3, 134.9, 134.1, 132.2, 128.9, 128.6, 126.3, 125.8, 124.5, 122.8, 46.8. HR-MS (ESI): Calcd for $C_{14}H_{11}BrN^+$ [M - Cl]$^+$ 272.0069 and 274.0049; found 272.0068 and 274.0049.

Synthesis of 5-methylphenanthridin-6(5*H*)-one (**13a**)

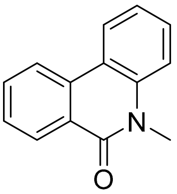

13a

Phenanthridin-6(5*H*)-one (**3h**, 0.5 g, 2.6 mmol) was dissolved in anhydrous THF (30 mL), and cesium carbonate (Cs_2CO_3, 0.85 g, 2.6 mmol) was added. The mixture was refluxed for 6 h, then was concentrated by vacuum. The residue was dissolved in water (40 mL) and was extracted with CH_2Cl_2 (50 mL×3). The combined organic phase was washed with brine (40 mL), dried over anhydrous Na_2SO_4, and concentrated. The residue was purified by column chromatography on

silica gel with petroleum ether / ethyl acetate (6:1，v/v) to give 5-methylphenanthridin-6(5*H*)-one (**13a**, 0.47 g, 89%) as a white solid, mp 105–106 °C (lit.[10] mp 106–107 °C). ^{1}H NMR (400 MHz, $CDCl_3$) *δ* 8.56 (d, *J* = 8.0 Hz, 1H), 8.32 – 8.25 (m, 2H), 7.76 (t, *J* = 7.6 Hz, 1H), 7.65 – 7.50 (m, 2H), 7.43 (d, *J* = 8.4 Hz, 1H), 7.33 (t, *J* = 7.6 Hz, 1H), 3.82 (s, 3H).

Compounds **13b~e** were prepared like **13a**

5-Allylphenanthridin-6(5*H*)-one (**13b**)

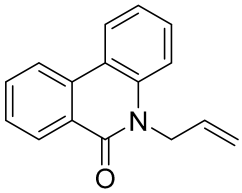

13b

Yellow solid; mp 96–97 °C (lit.[11] mp 97–98 °C); yield 73%. ^{1}H NMR (400 MHz, $CDCl_3$) *δ* 8.64 – 8.50 (m, 1H), 8.35 – 8.24 (m, 2H), 7.83 – 7.73 (m, 1H), 7.65 – 7.57 (m, 1H), 7.55 – 7.48 (m, 1H), 7.44 – 7.36 (m, 1H), 7.35 – 7.29 (m, 1H), 6.13 – 5.95 (m, 1H), 5.29 – 5.21 (m, 1H), 5.20 – 5.12 (m, 1H), 5.10 – 5.04 (m, 2H).

5-(prop-2-yn-1-yl)Phenanthridin-6(5*H*)-one (**13c**)

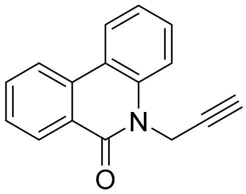

13c

Yellow solid; mp 167–168 °C (lit.[11] mp 168–170 °C); yield 80%. ^{1}H NMR (400 MHz, $CDCl_3$) *δ* 8.63 – 8.49 (m, 1H), 8.36 – 8.21 (m, 2H), 7.82 – 7.71 (m, 1H), 7.66 – 7.53 (m, 3H), 7.35 (ddd, *J* = 8.2, 5.1, 3.2 Hz, 1H), 5.21 (s, 2H), 2.26 (t, *J* = 2.4 Hz, 1H).

5-Isopropylphenanthridin-6(5*H*)-one (**13d**)

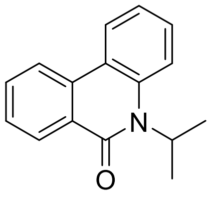

13d

Yellow solid; m.p. 99–100 °C (lit.[12] mp 99–101 °C); yield 55%. ^{1}H NMR (400 MHz, $CDCl_3$) *δ* 8.65 – 8.41 (m, 1H), 8.27 – 8.11 (m, 2H), 7.74 – 7.41 (m, 4H), 7.30 – 7.17 (m, 1H), 6.08 – 4.95 (m, 1H), 1.70 (d, *J* = 7.0 Hz, 6H).

5-Benzylphenanthridin-6(5*H*)-one (**13e**)

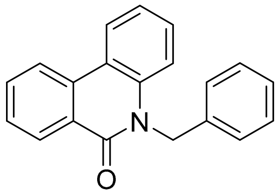

13e

Yellow solid; m.p. 115–116 °C (lit.[12] mp 112–113 °C); yield 65%. ^{1}H NMR (400 MHz, $CDCl_3$) *δ* 8.70 – 8.63 (m, 1H), 8.36 – 8.27 (m, 2H), 7.86 – 7.79 (m, 1H), 7.69 – 7.62 (m, 1H), 7.45 – 7.38 (m, 1H), 7.37 – 7.25 (m, 7H), 5.70 (s, 2H).

Synthesis of *N*-([1,1'-biphenyl]-2-yl)-2-chloroacetamide (**15**)

$ClCOCH_2Cl$ (2.6 mL, 32.5 mmol) was added dropwise to a solution of 2-aminobiphenyl (**14**, 5 g，29.6 mmol) and Et_3N (4.5 mL，32.5 mmol) in anhydrous CH_2Cl_2 (25 mL). The mixture was stirred at room temperature for 4 h, then poured into 50 mL of water, and extracted with CH_2Cl_2 (30 mL × 3). The organic layer was combined, washed with brine, dried over anhydrous sodium sulfate (Na_2SO_4), filtered, and then concentrated. The residue was recrystallized from ethanol to provide *N*-([1,1'-biphenyl]-2-yl)-2-chloroacetamide as a white solid (**15**, 6.8 g, 94%), ^{1}H NMR (400 MHz, $CDCl_3$) *δ* 8.46 (s, 1H), 8.36 (d, *J* = 8.2 Hz, 1H), 7.50 (dd, *J* = 10.0, 4.5 Hz, 2H), 7.46 – 7.36 (m, 4H), 7.30 (dd, *J* = 7.6, 1.6 Hz, 1H), 7.23 (td, *J* = 7.5, 1.0 Hz, 1H), 4.07 (s, 2H).

Synthesis of dibenzo[*b*,*d*]azepin-6-one (**16**)

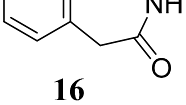

16

A mixture of compound **15** (5 g, 20 mmol) and aluminium chloride ($AlCl_3$, 6 g，44 mmol) in 1,3-dichlorbenzene (10 mL) was stirred at reflux for 9 h. The mixture was cooled to room temperature then poured into ice water (50 mL). After stirred several minutes, a white precipitate was liberated. The precipitate was collected by vacuum

filtration and washed several times with water. The filter cake was dried to give dibenzo[*b*,*d*]azepin-6-one as a white solid (**16**, 3.3 g, 79%), mp 230–231 °C (lit.[1] mp 228–230 °C). ^{1}H NMR (400 MHz, DMSO-d_6) δ 10.07 (s, 1H), 7.65 (d, J = 7.5 Hz, 1H), 7.62 – 7.57 (m, 1H), 7.48 – 7.37 (m, 4H), 7.27 (t, J = 7.3 Hz, 1H), 7.20 (d, J = 8.0 Hz, 1H), 3.43 (d, J = 12.1 Hz, 1H), 3.32 (d, J = 12.5 Hz, 1H).

Compounds **17** were prepared like **13a**

5-Methyldibenzo[*b*,*d*]azepin-6-one (**17**)

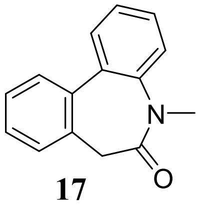
17

White solid; mp 157–158 °C (lit.[13] mp 156–157 °C); yield 89%. ^{1}H NMR (400 MHz, $CDCl_3$) δ 7.58 (dd, J = 7.6, 1.5 Hz, 2H), 7.50 – 7.37 (m, 4H), 7.37 – 7.28 (m, 2H), 3.62 (d, J = 12.7 Hz, 1H), 3.42 (d, J = 12.7 Hz, 1H), 3.33 (s, 3H).

Synthesis of 5-methyldibenzo[*b*,*d*]azepine (**18**)

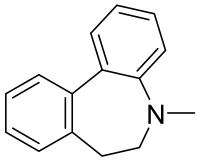
18

To a solution of **17** (1.0 g, 4.5 mmol) in dry THF (50 mL) was added $LiAlH_4$ (0.28 g, 7 mmol) in several portions at ice bath. Then the reaction mixture was refluxed for 4 h, and quenched with water (1 mL), and filtered, the filtrate was concentrated. The residue was purified by column chromatography on silica gel with petroleum ether / ethyl acetate (10:1， v/v) to give 5-methyldibenzo[*b*,*d*]azepine (**18**, 0.89 g, 95%) as a white solid, mp 53–54 °C. ^{1}H NMR (400 MHz, $CDCl_3$) δ 7.42 – 7.32 (m, 4H), 7.32 – 7.28 (m, 1H), 7.27 – 7.23 (m, H), 7.15 – 7.02 (m, 2H), 3.46 (t, J = 6.6 Hz, 2H), 2.78 (s, 3H), 2.73 (t, J = 6.6 Hz, 2H).

2. The detailed biological assay methods[14]

To prepare the compound solutions, the compound was first dissolved within a suitable amount of DMF and diluted with water containing 0.1% TW-80 to make a concentration of 500 $mg \cdot L^{-1}$, and the aqueous solution was diluted to 100 $mg \cdot L^{-1}$.

Protective Effect of Compounds against TMV in Vivo.

The compound solution was smeared on growing *Nicotiana. tabacum* L. leaves (at least 3 leaves) of the same age. In another pot, the leaves were smeared with the solvent as a control. After 12 h, the leaves were inoculated with TMV with the juice-leaf rubbing method and then washed with water. The total local lesion numbers appearing on the leaves 3–4 days after inoculation were recorded. The experiment was conducted with three replicates for each compound.

Inactivation Effect of Compounds against TMV .

To test viral inhibition, equal volumes of the virus and the compound solution were mixed together for 30 min. The mixture was then inoculated into the growing *N. tabacum* L leaves of the same age, and another pot was inoculated with the mixture of solvent and the virus as the control. The local lesion numbers were recorded 3–4 days after inoculation.

Curative Effect of Compounds against TMV

TMV (concentration of 6.0×10^{-3} $mg \cdot L^{-1}$) was inoculated on the growing leaves of *N. tabacum* L. of the same age. Then, the leaves were washed with

water and dried. After about 6h, the compound solution was smeared on the inoculated leaves, while inoculated leaves in another pot were smeared with the solvent as a control. The local lesion numbers were recorded 3–4 days after inoculation. All assays were conducted using three replicates for each compound. The inhibition rates of the compound were calculated according to the following formula ("av" means average, and " controls" means not treated with compound): *inhibition rate (%) = [(av local lesion number of control − av local lesion number of drug treated)/av local lesion number of control] × 100%.*

3. Mode of action of anti-TMV studies

3.1 TMV virus purification

According to the method provided by Leberman[15], we purified tobacco mosaic virus (TMV). TMV-infected tobacco leaves were grounded with the pestle in the ice-cold mortar with 25 mL 0.2 M phosphorous buffer (PB) buffer (pH 7.0) including 250 μL *β*-mercaptoethanol (1%, v/v). Two-layer screen cloth was used to filter tobacco debris to collect the flowthrough. The flowthrough was mixed with 8% *n*-butyl alcohol and gently mixed for 15 min for chlorophyll withdraw. After centrifugation at 10,000 rmp for 15 min, the supernatant was mixed with NaCl (4%, w/v) and PEG 6000 (4%) on ice for virus precipitation in 6 hours. After centrifugation at 10,000 rmp for 20 min, the precipitate was fully dissolved in 5 mL 0.01 M PB buffer (pH 7.0) for 2 hours. After centrifugation at 10,000 rmp for 20 min, the supernatant was then stirred again with NaCl (4%, w/v) and PEG6000 (4%, w/v) overnight on ice for virus precipitation. After

centrifugation at 10,000 rmp for 20 min, the precipitate was dissolved in 2 mL 0.01 M PB buffer (pH 7.0) for 6 hours. After centrifugation at 10,000 rmp for 5 min, the white supernatant was the TMV solution. The TMV purity was confirmed according to the specific ratio of A_{280}/A_{260} (0.84) and A_{260}/A_{248} (1.09). The virus concentration was calculated according to the formula $C_{(mg/mL)}=A_{260}/3.1$. The TMV solution was kept in storage at 4 °C.

3.2 TMV CP purification

The TMV capsid protein (TMV CP) was purified according to the following procedure from Fraenkelconrat[16]. 1 mL TMV virus suspension (5 mg/mL) was mixed with 2 mL glacial acetic acid to break up at 4 °C for 2 h. After centrifugation at 10,000 rmp for 30 min, the supernatant was transferred into a new tube. Subsequently, 3 mL TMV sterile water was added. The mixture was finally transferred into a dialysis bag for TMV CP precipitation. After three times of water exchange, we could see a lump of CP aggregates in the bag. After centrifugation at 12,000 rmp for 30 min, the TMV CP aggregates were collected and dissolved with 50 μL 0.1 M phosphate buffer (pH 7.0). After centrifugation at 4000 rpm for 15 min, the supernatant was obtained as the TMV CP. The concentration was determined according to the formula $C(mg/mL)=A_{282}/1.27$. The purified TMV CP was kept in storage at 4 °C.

3.3 TMV RNA purification

TMV RNA was purified using the method of phenol:chloroform:isopentanol (25:24:1).[15] 1 mL TMV virus suspension (20

mg/mL) was mixed with 1 mL phenol: chloroform: isopentanol (25:24:1). After centrifugation at 12,000 rpm for 20 min at 4 °C, the upper phase was transferred into a new RNase-free tube and the mixed with 1 mL chloroform to remove excess phenol. After centrifugation at 12,000 rpm for 20 min at 4 °C, the upper phase was transferred into a new RNase-free tube. 100 μL sodium acetate (3 M, pH 5.2) and 2.2 mL of absolute alcohol was added and kept in -20 °C for 30 min. After centrifugation at 12,000 rpm for 20 min at 4 °C, the RNA precipitate was washed with 70% alcohol for two times. The final RNA precipitate was dissolved in RNase-free water and kept in storage in -80 °C. The concentration of TMV RNA was determined using nanodrop2000.

3.4 In vitro 20S disk inhibition reaction

For drug test, in vitro 20S disk inhibition reaction was performed at 20 °C for 12 h after adding 9.8 μL TMV capsid proteins (20 mg/mL) and 0.2 μL DMSO or drug (10 nmol/mL, 0.2 μL in DMSO). After the treatment, the sample was used for transmission electron microscope (TEM) characterization.

3.5 In vitro TMV reconstitution reaction

Before the assembly of TMV nanorods, 20S disk was prepared by incubating TMV capsid proteins (20 mg/mL) in 0.1 M phosphate buffer (pH 7.0) at 20 °C for 12 h. The TMV nanorod assembly reaction was performed by mixing 5 μL phosphate buffer (0.1 mol/L, pH 7.0), 4 μL 20S disk (20 mg/mL) and 1 μL TMV RNA (200 ng/μL). The assembly reaction was incubated at 20 °C for 12 h. For drug test, in vitro TMV reconstitution inhibition reaction was performed

by adding 4.8 μL phosphate buffer (0.1 mol/L, pH 7.0), 4 μL 20S disk (20 mg/mL), 1 μL TMV RNA (200 mg/mL) and 0.2 μL DMSO or drug (10 nmol/mL, 0.2 μL in DMSO).

3.6 Transmission electron microscope (TEM) characterization

The sample was dropped onto a carbon-coated copper grid. After 2 min, the sample on the copper grid was removed with the filter paper and the copper grid was immersed with a 2% uranyl acetate solution for 2 min. The sample was imaged by the JEM-2100F transmission electron microscope operated at 200 kV.

4. ^{1}H NMR and ^{13}C NMR spectra for new compounds.

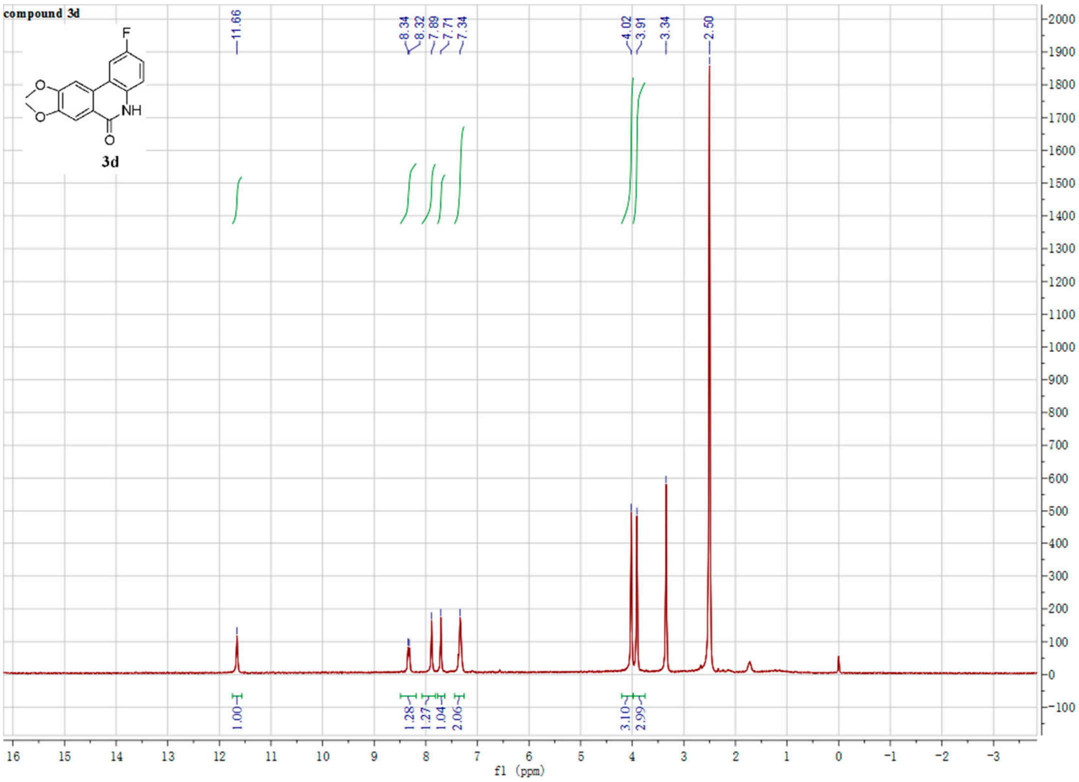

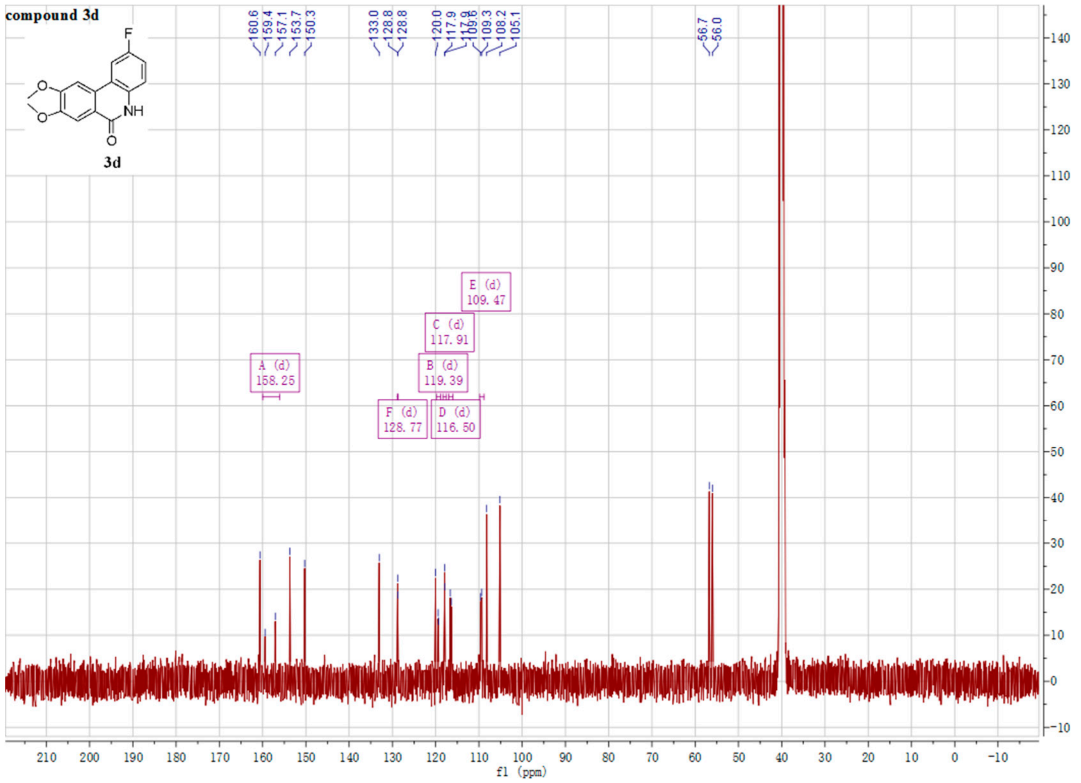

compound 3d
3d
160.6
159.4
157.1
153.7
150.3
133.0
128.8
128.8
120.0
117.9
109.3
108.2
105.1
56.7
56.0
A (d) 158.25
B (d) 119.39
C (d) 117.91
D (d) 116.50
E (d) 109.47
F (d) 128.77
f1 (ppm)

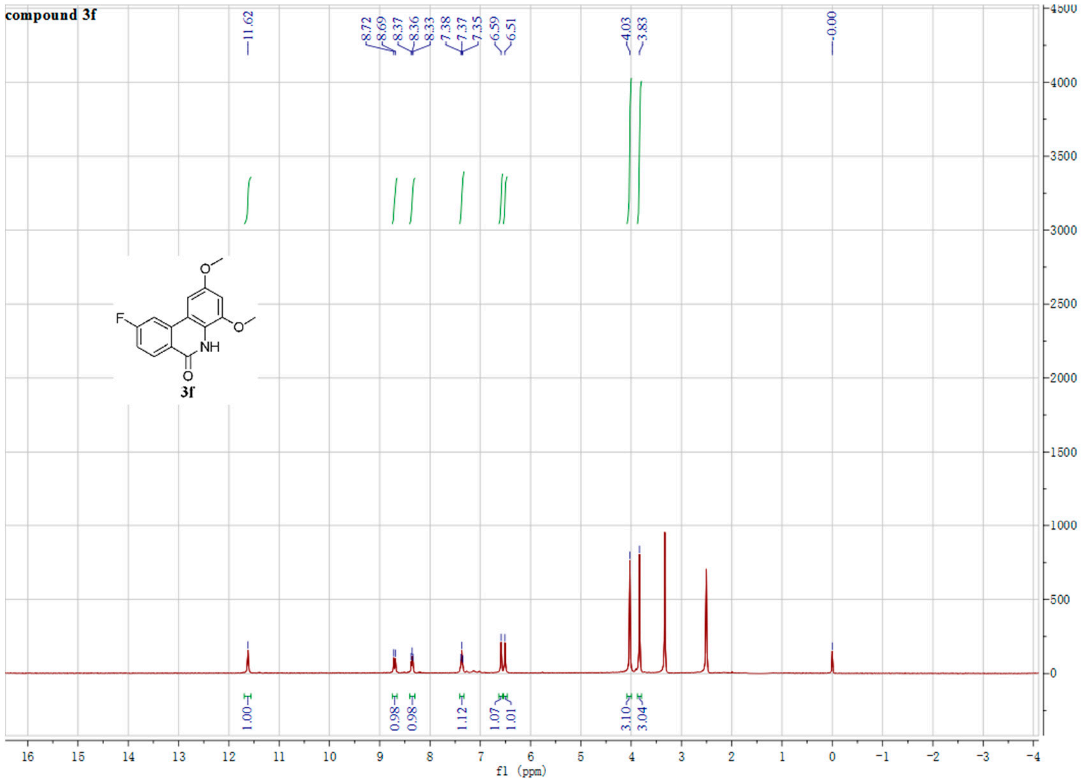

compound 3f
3f
11.62
8.72
8.69
8.37
8.36
8.33
7.38
7.37
7.35
6.59
6.51
4.03
3.83
0.00
1.00
0.98
0.98
1.12
1.07
1.01
3.10
3.04
f1 (ppm)

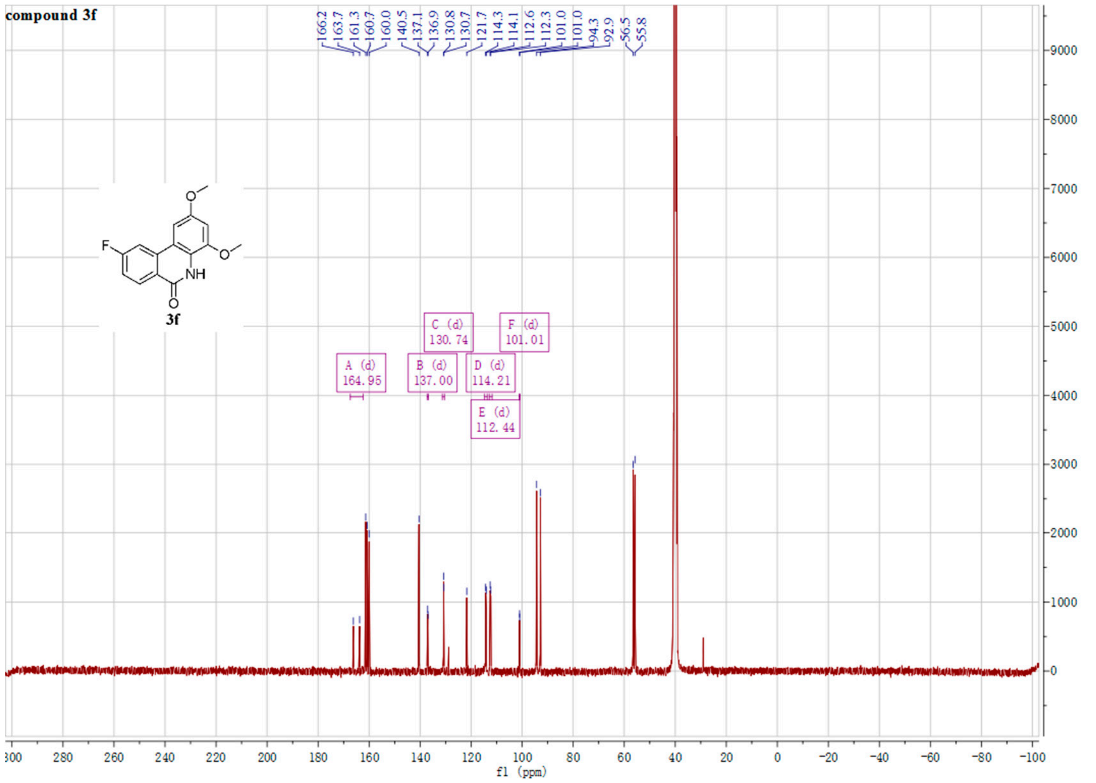
compound 3f
166.2
163.7
161.3
160.7
160.0
140.5
137.1
136.9
130.8
130.7
121.7
114.3
114.1
112.6
112.3
101.0
101.0
94.3
92.9
56.5
55.8
F
NH
O
3f
A (d) 164.95
B (d) 137.00
C (d) 130.74
D (d) 114.21
E (d) 112.44
F (d) 101.01
f1 (ppm)

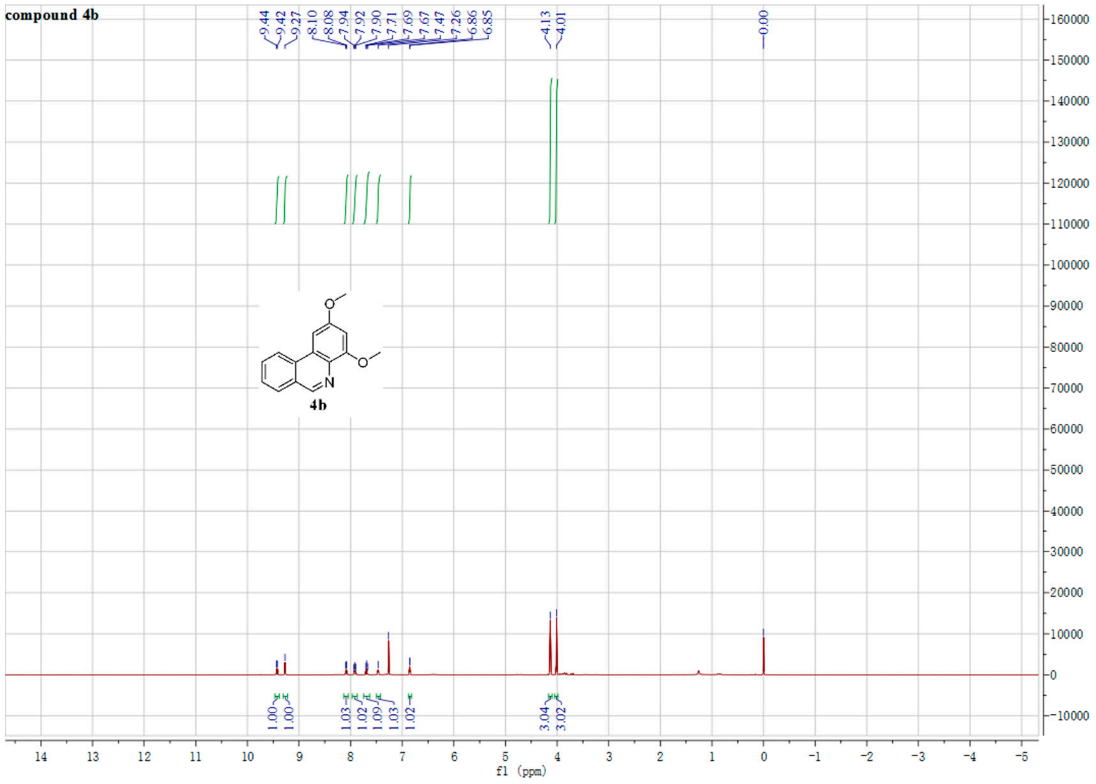
compound 4b
9.44
9.42
9.27
8.10
8.08
7.94
7.92
7.90
7.71
7.69
7.67
7.47
7.26
6.86
6.85
4.13
4.01
0.00
N
4b
1.00
1.00
1.03
1.02
1.09
1.03
1.02
3.04
3.02
f1 (ppm)

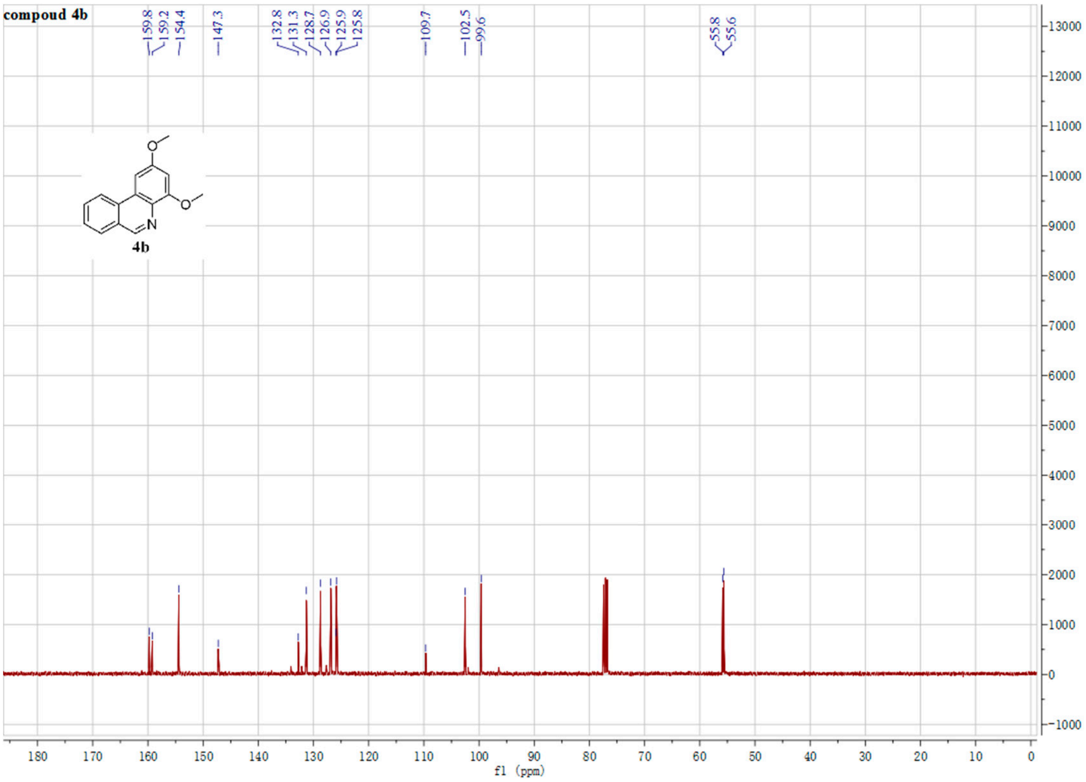
compoud 4b
159.8
159.2
154.4
147.3
132.8
131.3
128.7
126.9
125.9
125.8
109.7
102.5
99.6
55.8
55.6
4b
f1 (ppm)

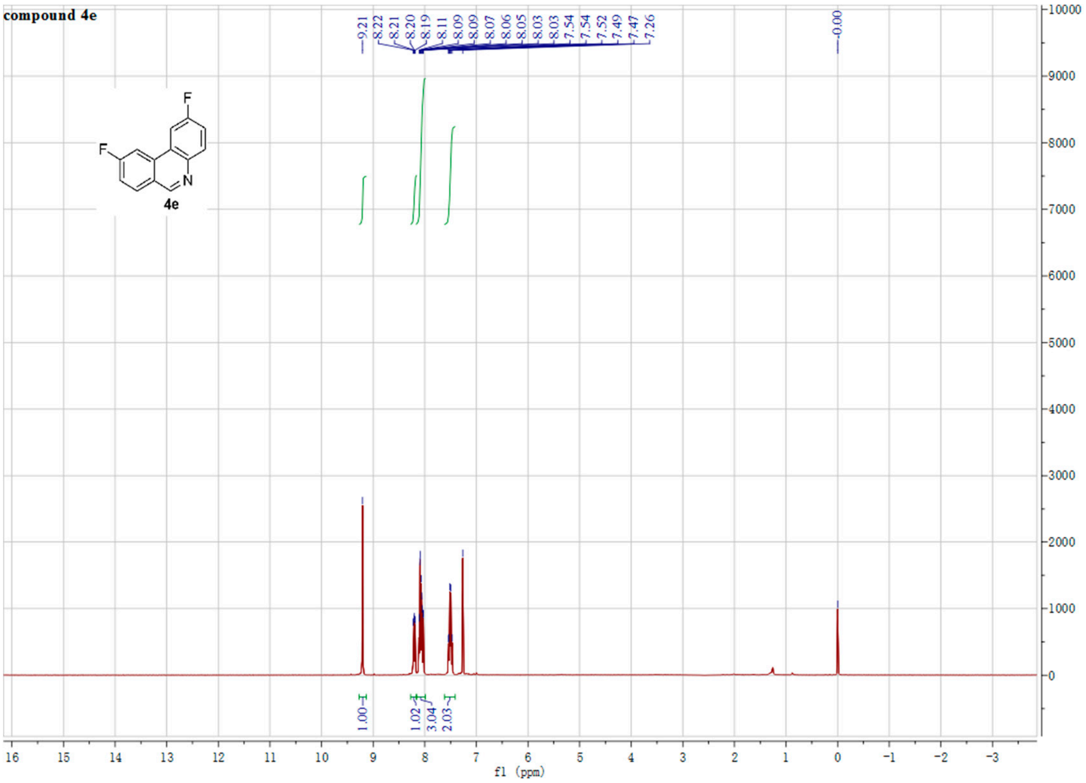
compound 4e
9.21
8.22
8.21
8.20
8.19
8.11
8.09
8.09
8.07
8.06
8.05
8.03
8.03
7.54
7.54
7.52
7.49
7.47
7.26
-0.00
4e
1.00
1.02
3.04
2.03
f1 (ppm)

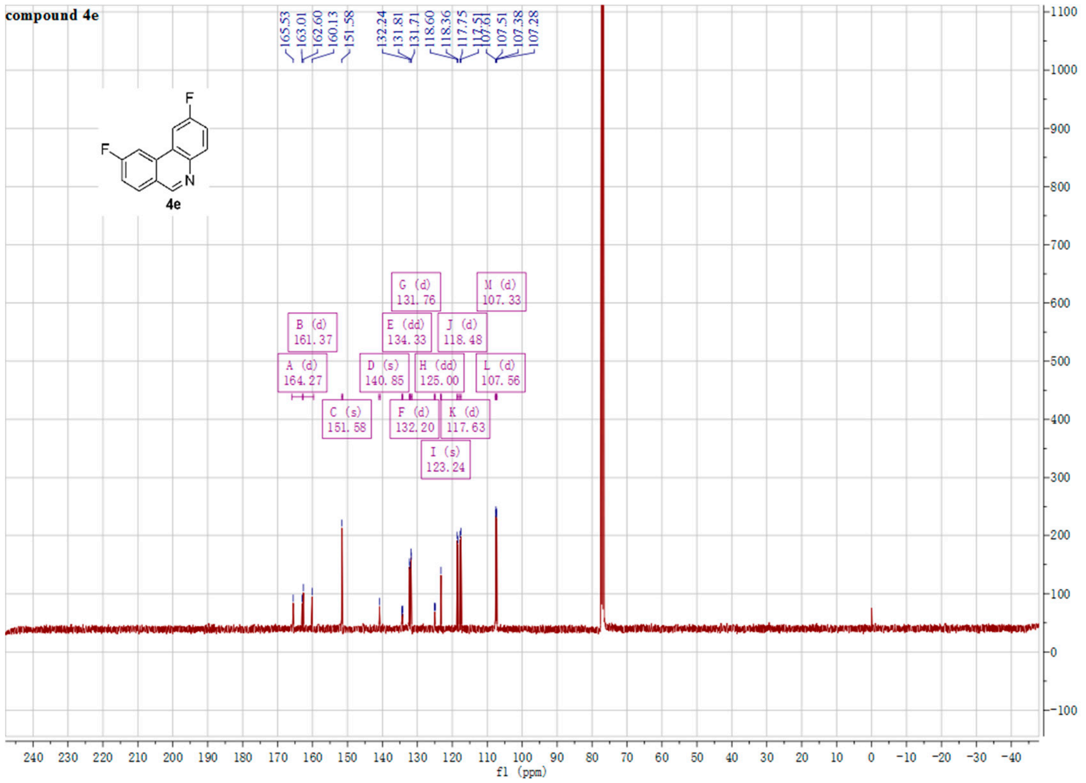
compound 4e
4e
165.53
163.01
162.60
160.13
151.58
132.24
131.81
131.71
118.60
118.36
117.75
107.51
107.38
107.28
A (d) 164.27
B (d) 161.37
C (s) 151.58
D (s) 140.85
E (dd) 134.33
F (d) 132.20
G (d) 131.76
H (dd) 125.00
I (s) 123.24
J (d) 118.48
K (d) 117.63
L (d) 107.56
M (d) 107.33
f1 (ppm)

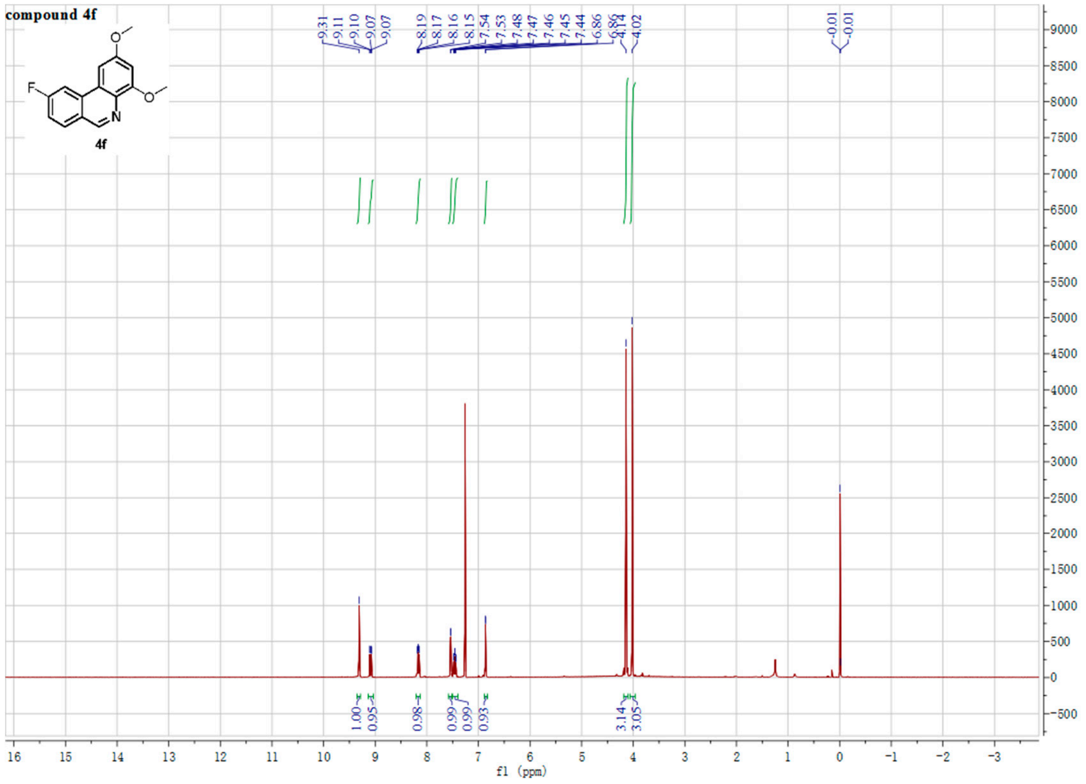
compound 4f
4f
9.31
9.11
9.10
9.07
9.07
8.19
8.17
8.16
8.15
7.54
7.53
7.48
7.47
7.46
7.45
7.44
6.86
4.14
4.02
-0.01
-0.01
1.00
0.95
0.98
0.99
0.99
0.93
3.14
3.05
f1 (ppm)

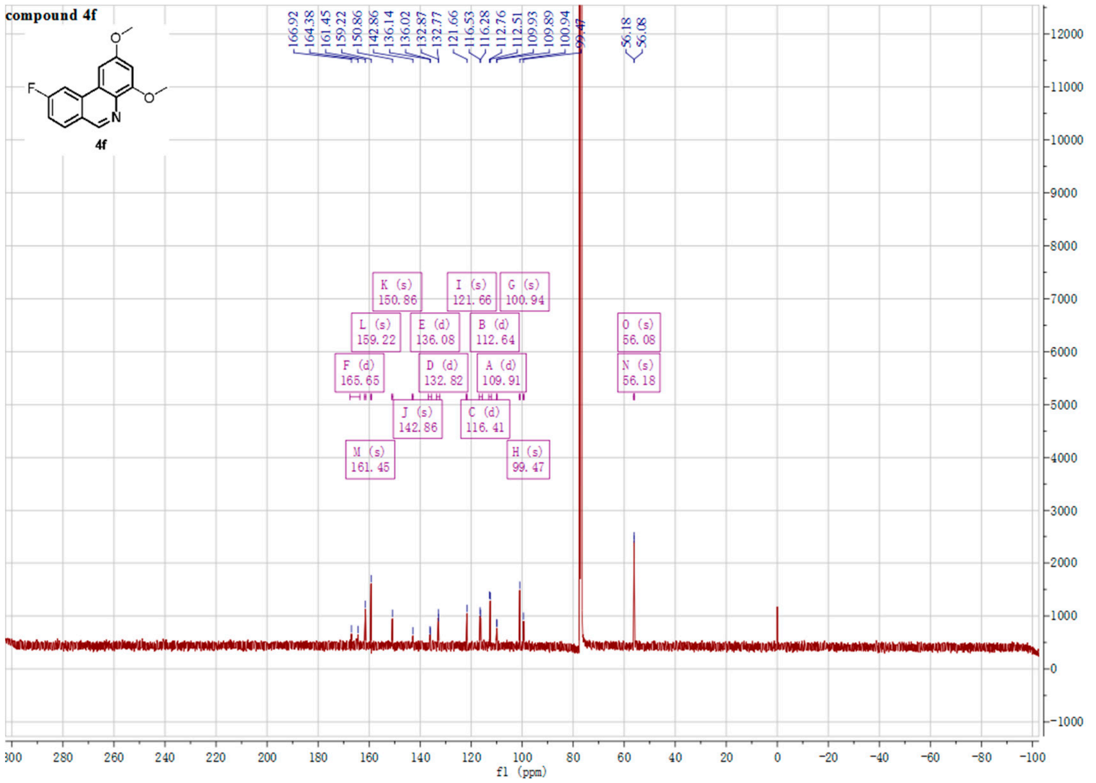
compound 4f
F
N
4f
166.92
164.38
161.45
159.22
150.86
142.86
136.14
136.02
132.87
132.77
121.66
116.53
116.28
112.76
112.51
109.93
109.89
100.94
99.47
56.18
56.08
K (s) 150.86
I (s) 121.66
G (s) 100.94
L (s) 159.22
E (d) 136.08
B (d) 112.64
O (s) 56.08
F (d) 165.65
D (d) 132.82
A (d) 109.91
N (s) 56.18
J (s) 142.86
C (d) 116.41
M (s) 161.45
H (s) 99.47
f1 (ppm)

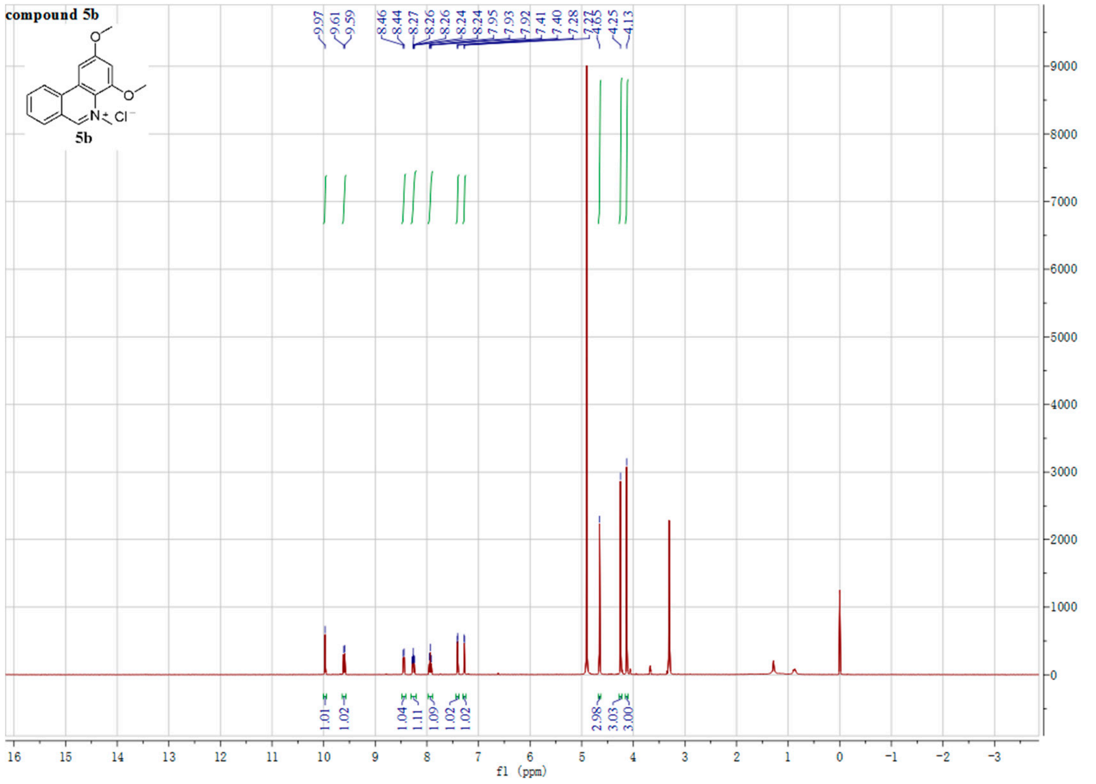
compound 5b
N
Cl
5b
9.97
9.61
9.59
8.46
8.44
8.27
8.26
8.24
8.24
7.95
7.93
7.92
7.41
7.40
7.28
7.27
4.65
4.25
4.13
1.01
1.02
1.04
1.11
1.09
1.02
1.02
2.98
3.03
3.00
f1 (ppm)

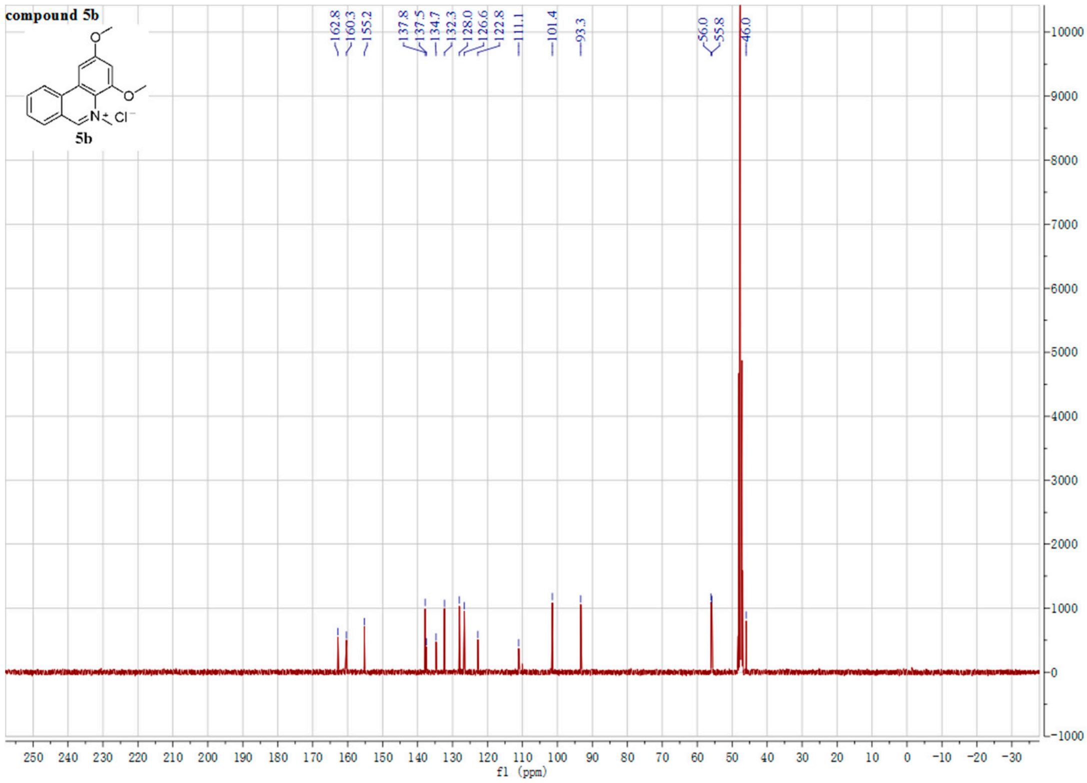

compound 5b
5b
162.8
160.3
155.2
137.8
137.5
134.7
132.3
128.0
126.6
122.8
111.1
101.4
93.3
56.0
55.8
46.0
f1 (ppm)

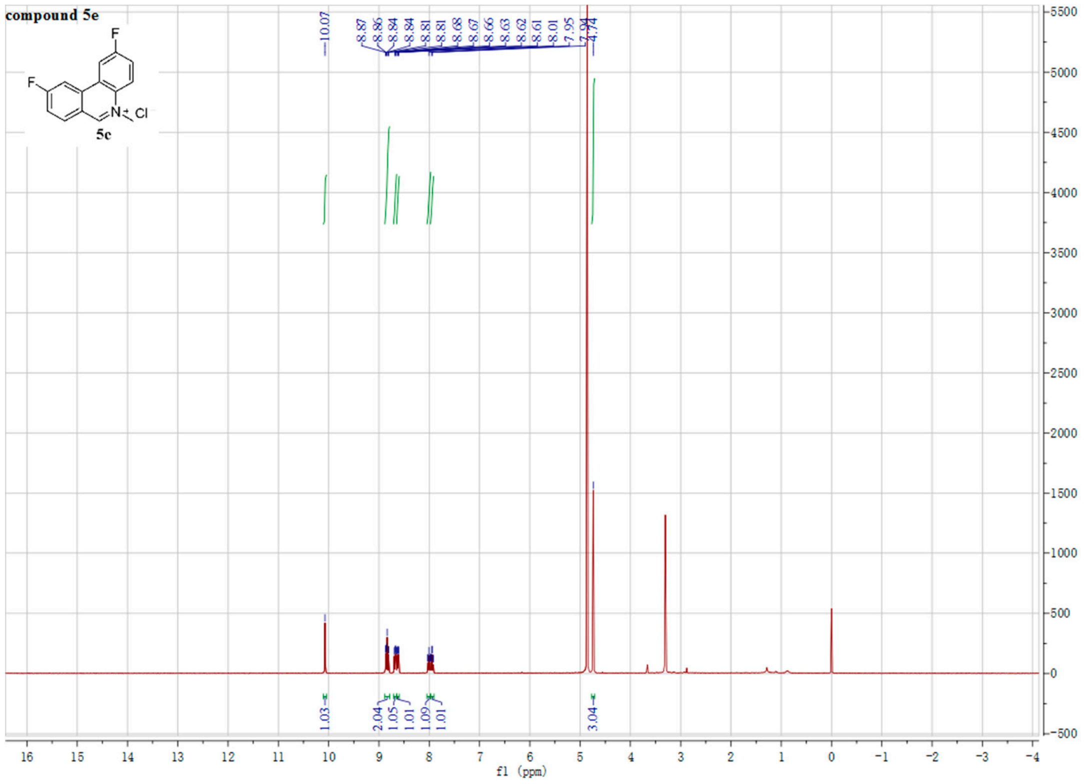

compound 5e
5c
10.07
8.87
8.86
8.84
8.84
8.81
8.81
8.68
8.67
8.66
8.63
8.62
8.61
8.01
7.95
7.94
4.74
1.03
2.04
1.05
1.01
1.09
1.01
3.04
f1 (ppm)

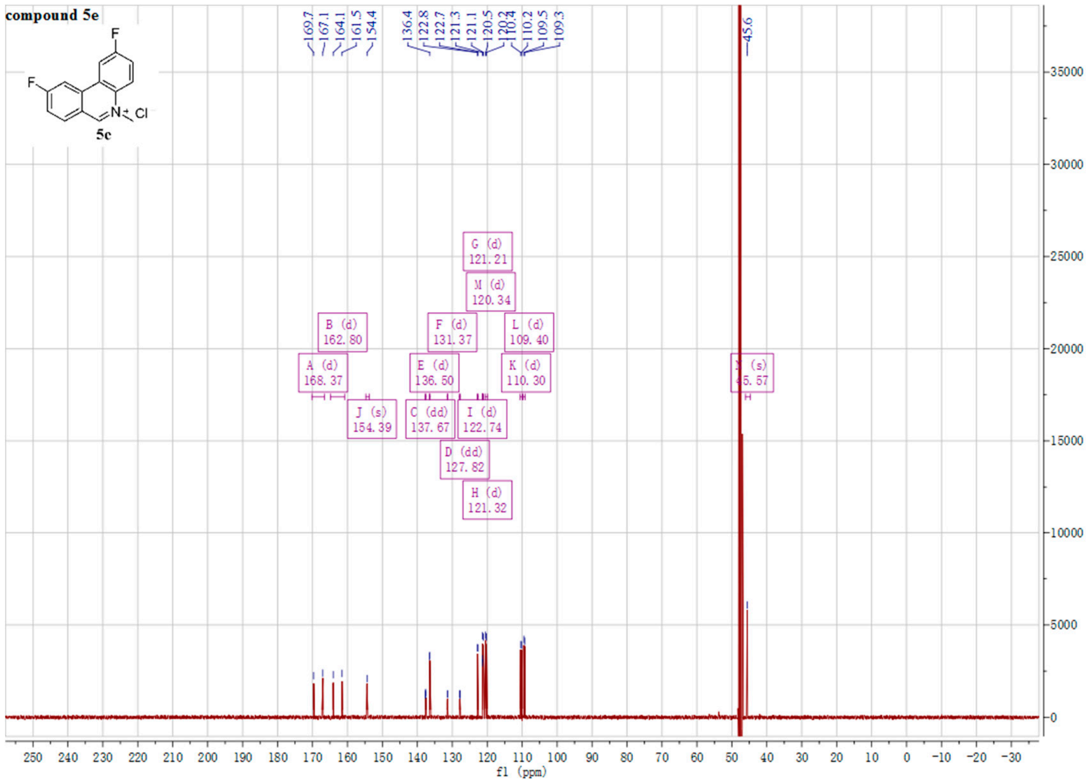
compound 5e
5c
169.7
167.1
164.1
161.5
154.4
136.4
122.8
122.7
121.3
121.1
120.5
120.2
110.4
110.2
109.5
109.3
45.6
f1 (ppm)

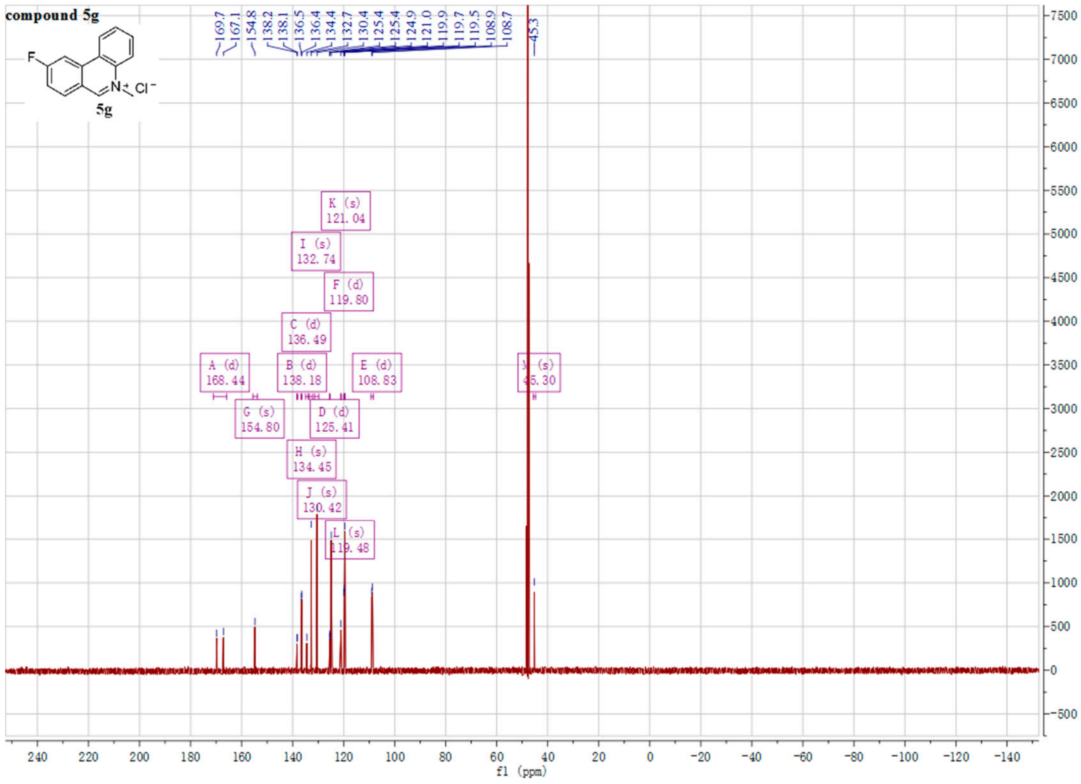
compound 5g
5g
169.7
167.1
154.8
138.2
138.1
136.5
136.4
134.4
132.7
130.4
125.4
125.4
124.9
121.0
119.9
119.7
119.5
108.9
108.7
45.3
f1 (ppm)

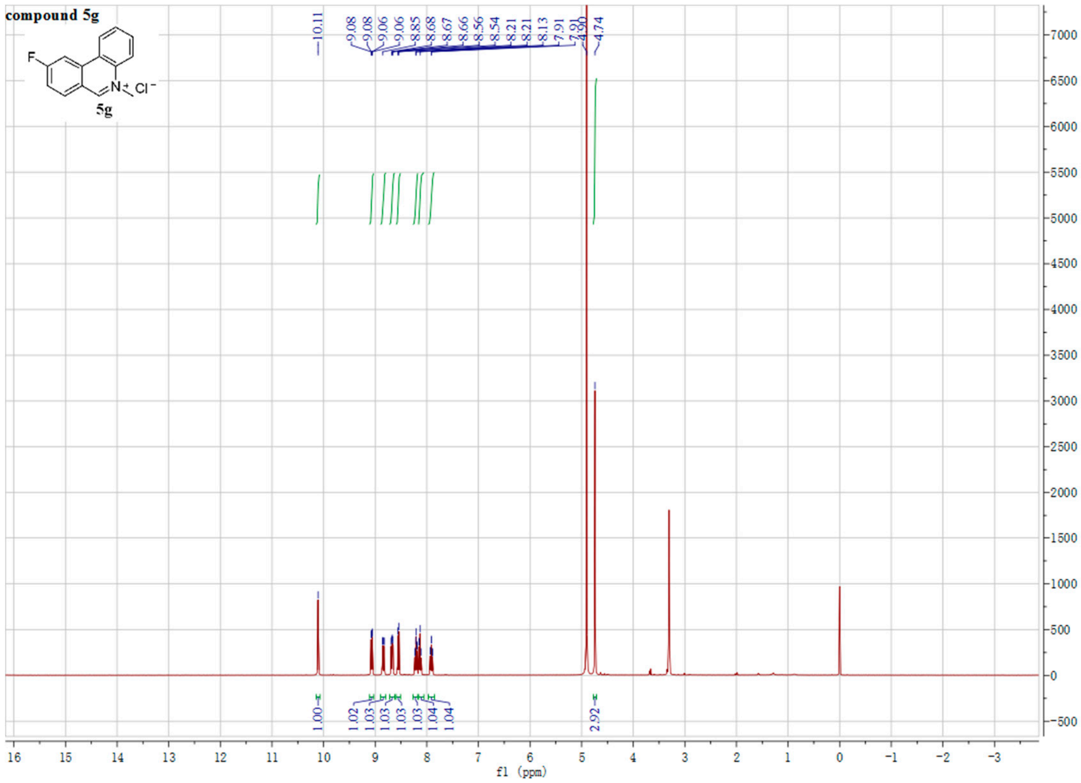
compound 5g
5g
10.11
9.08
9.08
9.06
9.06
8.85
8.68
8.67
8.66
8.56
8.54
8.21
8.21
8.13
7.91
4.90
4.74
1.00
1.02
1.03
1.03
1.03
1.03
1.04
1.04
2.92
f1 (ppm)

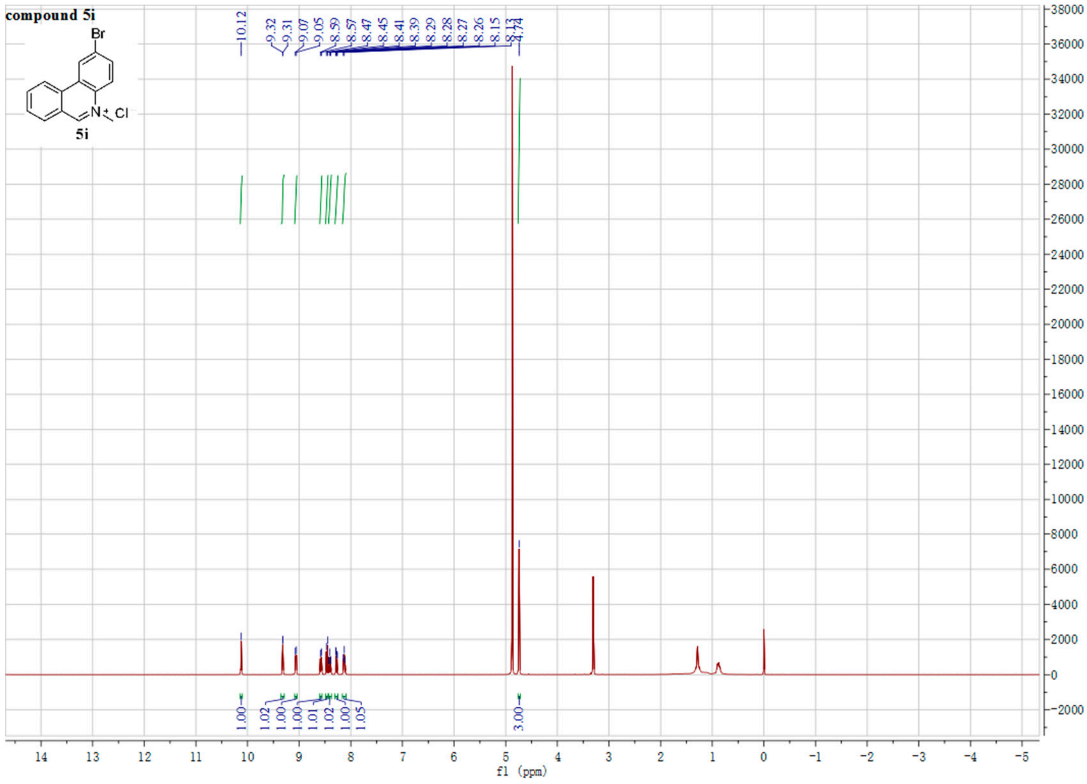
compound 5i
5i
10.12
9.32
9.31
9.07
9.05
8.59
8.57
8.47
8.45
8.41
8.39
8.29
8.28
8.27
8.26
8.15
8.13
4.74
1.00
1.02
1.00
1.00
1.01
1.02
1.00
1.05
3.00
f1 (ppm)

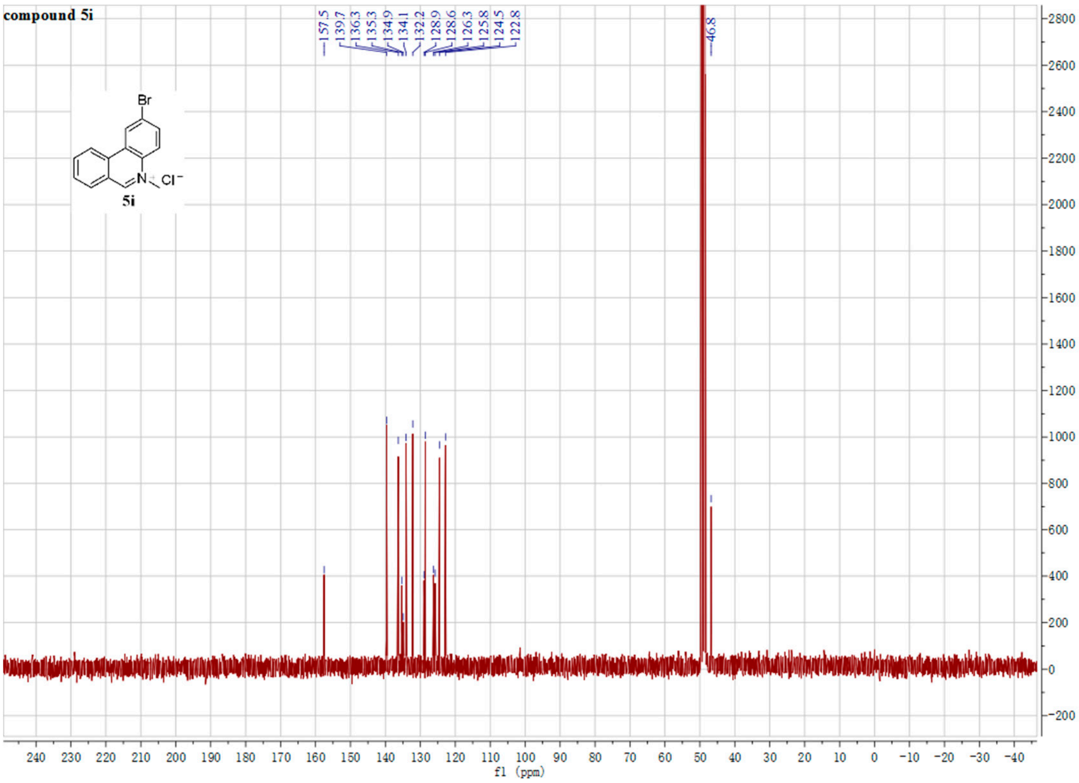

References

1. Bhakuni, B.S.; Kumar, A.; Balkrishna, S.J.; Sheikh, J.A.; Konar, S.; Kumar, S. Kotbu Mediated Synthesis of Phenanthridinones and Dibenzoazepinones. *Org. Lett.* **2012**, *14*, 2838-2841, doi:10.1021/ol301077y.
2. Meseroll, L.M.N.; McKee, J.R.; Zanger, M. Synthesis of 5,6-Dihydrophenanthridine (Dhpa) Sulfonamides and Subsequent Acid-Catalyzed Rearrangement to Diaryl Sulfones. *Synth. Commun.* **2011**, *41*, 2557-2568, doi:10.1080/00397911.2010.515329.
3. Pan, H.L.; Fletcher, T.L. 6(5h)-Phenanthridinones. III. Halo-6(5h)Phenanthridinones. *J. Heterocycl. Chem.* **1970**, *7*, 597-605, doi:doi:10.1002/jhet.5570070320.
4. Baechler, S.A.; Fehr, M.; Habermeyer, M.; Hofmann, A.; Merz, K.-H.; Fiebig, H.-H.; Marko, D.; Eisenbrand, G. Synthesis, Topoisomerase-Targeting Activity and Growth Inhibition of Lycobetaine Analogs. *Bioorg. Med. Chem.* **2013**, *21*, 814-823, doi:https://doi.org/10.1016/j.bmc.2012.11.011.
5. Fujita, R.; Yoshisuji, T.; Wakayanagi, S.; Wakamatsu, H.; Matsuzaki, H. Synthesis of 5(6h)-Phenanthridones Using Diels–Alder Reaction of 3-Nitro-2(1h)-Quinolones Acting as Dienophiles. *Chem. Pharm. Bull* **2006**, *54*, 204-208, doi:10.1248/cpb.54.204.
6. Hoffmann-Emery, F.; Jakob-Roetne, R.; Flohr, A.; Bliss, F.; Reents, R. Improved Synthesis of (S)-7-Amino-5h,7h-Dibenzo[B,D]Azepin-6-One, a Building Block for Γ-Secretase Inhibitors. *Tetrahedron Lett.* **2009**, *50*, 6380-6382, doi:10.1016/j.tetlet.2009.08.090.
7. Chen, Y.-F.; Wu, Y.-S.; Jhan, Y.-H.; Hsieh, J.-C. An Efficient Synthesis of (Nh)-Phenanthridinones Via Ligand-Free Copper-Catalyzed Annulation. *Org. Chem. Front.* **2014**, *1*, 253-257, doi:10.1039/C3QO00082F.

8. Chen, W.; Chen, C.; Chen, Y.; Hsieh, J. Hydride-Induced Anionic Cyclization: An Efficient Method for the Synthesis of 6-H-Phenanthridines Via a Transition-Metal-Free Process. *Org. Lett.* **2015**, *17*, 1613-1616, doi:10.1021/acs.orglett.5b00544.
9. Liu, F.; Venter, H.; Bi, F.; Semple, S.J.; Liu, J.; Jin, C.; Ma, S. Synthesis and Antibacterial Activity of 5-Methylphenanthridium Derivatives as Ftsz Inhibitors. *Bioorg. Med. Chem. Lett.* **2017**, *27*, 3399-3402, doi:https://doi.org/10.1016/j.bmcl.2017.06.005.
10. Ferraccioli, R.; Carenzi, D.; Motti, E.; Catellani, M. A Simple Catalytic Synthesis of Condensed Pyridones from O-Bromoarylcarboxamides Involving Ipso Substitution Via Palladacycles. *J. Am. Chem. Soc.* **2006**, *128*, 722-723, doi:10.1021/ja0566127.
11. Cookson, R.F.; James, J.W.; Rodway, R.E.; Simmonds, R.G. Synthesis of Some Phenanthridone Derivatives. *J. Heterocycl. Chem.* **1972**, *9*, 475-480, doi:doi:10.1002/jhet.5570090303.
12. Lu, C.; Dubrovskiy, A.V.; Larock, R.C. Palladium-Catalyzed Annulation of Arynes by O-Halobenzamides: Synthesis of Phenanthridinones. *J. Org. Chem.* **2012**, *77*, 8648-8656, doi:10.1021/jo3016192.
13. Tabata, H.; Suzuki, H.; Akiba, K.; Takahashi, H.; Natsugari, H. Atropisomeric Properties of 7-, 8-, and 9-Membered-Ring Dibenzolactams: Conformation, Thermal Stability, and Chemical Reactivity. *J. Org. Chem.* **2010**, *75*, 5984-5993, doi:10.1021/jo1013383.
14. Hu, Z.; Wang, Z.; Liu, Y.; Wang, Q. Leveraging Botanical Resources for Crop Protection: The Isolation, Bioactivity and Structure–Activity Relationships of Lycoris Alkaloids. *Pest Manag. Sci.* **2018**, *74*, 2783-2792, doi:10.1002/ps.5065.
15. Leberman, R. The Isolation of Plant Viruses by Means of "Simple" Coacervates. *Virology* **1966**, *30*, 341-347, doi:https://doi.org/10.1016/0042-6822(66)90112-7.
16. Fraenkel-Conrat, H.; Williams, R.C. Reconstitution of Active Tobacco Mosaic Virus from Its Inactive Protein and Nucleic Acid Components. *Proc. Natl. Acad. Sci. USA* **1955**, *41*, 690-698, doi:10.1073/pnas.41.10.690.
